# Metabolic reprogramming tailors T cell immunity in sepsis

**DOI:** 10.3389/fimmu.2025.1679493

**Published:** 2026-01-16

**Authors:** Di Xian, Feng Chen, Bing Liu, Lei Wang

**Affiliations:** Emergency Department, Sichuan Provincial People’s Hospital, School of Medicine, University of Electronic Science and Technology of China, Chengdu, China

**Keywords:** immunosuppression, metabolic reprogramming, sepsis, T-cell immunity, therapeutic strategies

## Abstract

Sepsis is a life-threatening organ dysfunction caused by a dysregulated host response to infection, characterized by persistently high morbidity and mortality. Current treatment strategies have limitations, particularly the persistence of an immunosuppressed state. Recent studies have revealed that sepsis not only causes immune system dysregulation but also leads to metabolic disturbances, specifically metabolic reprogramming in T cells—a field still in its early stages. This review systematically explores the mechanisms of T-cell metabolic reprogramming in sepsis, including enhanced glycolysis, mitochondrial dysfunction, and dysregulated amino acid metabolism. It further analyzes how these alterations, mediated by signaling pathways such as HIF-1α, mTOR, and AMPK, as well as key metabolic enzymes, exacerbate T-cell exhaustion and immunosuppression. The article elaborates on the role of metabolic reprogramming in T-cell dysfunction and susceptibility to secondary infections, and summarizes potential therapeutic strategies targeting metabolic pathways—such as IL-7 therapy and IDO1 inhibitors—for restoring T-cell function, offering new directions for sepsis immunotherapy.

## 1 Introduction

Sepsis is a life-threatening organ dysfunction caused by a dysregulated host response to infection (1). It encompasses a spectrum of disease severity ranging from bacteremia to septic shock and represents a systemic inflammatory response syndrome triggered by infection. If the condition worsens, it can progress to septic shock, multiple organ dysfunction syndrome (MODS), and even death (2, 3). The incidence and mortality rates of sepsis remain high globally. Each year, over 19 million new cases are reported worldwide, with approximately 6 million deaths. The case fatality rate and disability rate exceed 50%, and the mortality rate is over 25% (4, 5). Moreover, sepsis leads to numerous adverse outcomes (6, 7). For instance, about 3 million survivors experience cognitive dysfunction, and approximately one-third of sepsis patients die within the first year after discharge (8, 9).

Current treatment strategies for sepsis primarily include antimicrobial therapy, source control of infection, and intravenous immunoglobulin administration (10–12). However, these treatment approaches have several limitations. Although clinical guidelines recommend administering appropriate intravenous antibiotics as soon as possible within one hour after the diagnosis of sepsis or septic shock, a meta-analysis has shown that the use of antimicrobial agents within one or three hours after diagnosis does not significantly reduce mortality (13). Additionally, the emergence of drug-resistant strains further restricts the effectiveness of antimicrobial therapy (14). The unclear etiology of infection in some sepsis patients poses challenges for targeted antimicrobial treatment. Meanwhile, fluid resuscitation may fail to fully correct microcirculatory disturbances and can lead to fluid overload.

In recent years, with advancements in critical care medicine, the 30-day mortality rate of sepsis has declined, yet long-term mortality continues to rise after the “acute event” (15). Many sepsis survivors later die from persistent, recurrent, hospital-acquired, and secondary infections (16). Despite advances in supportive care and early resuscitation, treatment options targeting the core pathology of sepsis—immune system paralysis—remain critically lacking. Among the factors contributing to this acquired immunodeficiency, the progressive functional exhaustion of T lymphocytes is considered a pivotal driver (17, 18). Immunomodulatory therapeutic strategies may help improve the long-term prognosis of sepsis patients, though their safety and efficacy require further validation in clinical trials.

Sepsis induces metabolic dysregulation in the body (19, 20). Patients often exhibit hyperglycemia and insulin resistance, likely to meet the energy demands of immune cells (21, 22). Additionally, suppressed fatty acid oxidation (FAO) and oxidative phosphorylation (OXPHOS) pathways in sepsis patients lead to impaired energy metabolism (23–25). Metabolic reprogramming plays a pivotal role in regulating immune responses by influencing immune cell activation, differentiation, proliferation, and effector functions. For instance, activated effector T cells demonstrate enhanced glycolysis through metabolic reprogramming, performing aerobic glycolysis even under oxygen-sufficient conditions (26, 27). Metabolites generated during this process can act as signaling molecules to modulate immune cell functions. For example, intermediates in the tricarboxylic acid (TCA) cycle—such as citrate and succinate—accumulate in pro-inflammatory cells, regulating other metabolic and functional pathways (28, 29).

This review aims to provide an in-depth exploration of the core mechanisms of T cell metabolic reprogramming in sepsis and its potential as a therapeutic target. We will systematically dissect the specific roles of glycolysis, mitochondrial oxidative phosphorylation, and key amino acid metabolism pathways in this process, elucidating how these metabolic alterations directly lead to T cell functional failure by influencing epigenetics and signal transduction. Furthermore, this article will critically evaluate novel therapeutic strategies based on metabolic modulation—such as IL-7 therapy and IDO1 inhibitors—focusing on how they can restore T cell immune function by “reprogramming” their metabolism. Finally, we will discuss the current limitations of research and future directions, aiming to provide new perspectives and a theoretical foundation for overcoming the challenge of immunosuppression in sepsis.

## 2 The role of metabolic reprogramming in sepsis

### 2.1 Metabolic reprogramming: shift from oxidative phosphorylation to glycolysis

**Figure 1:**
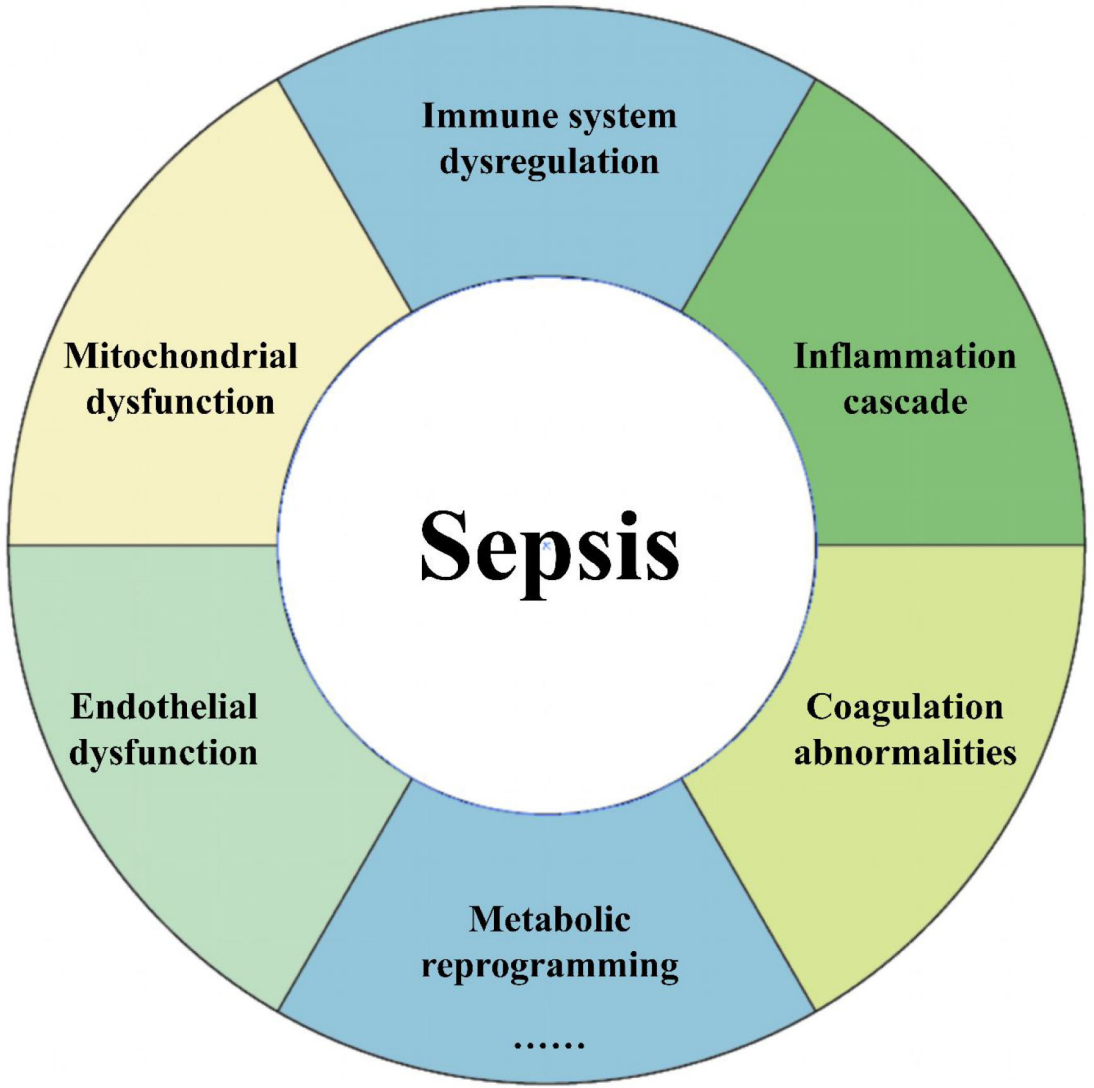
Pathological mechanisms of sepsis. Sepsis is a life-threatening condition that arises from a dysregulated immune response to infection, resulting in multiple organ dysfunction. The pathogenesis of sepsis is highly complex and multifaceted, extending beyond the virulence of the infecting pathogens. It involves intricate interactions among various immune, inflammatory, and coagulation pathways.

In sepsis, multiple mechanisms affect disease progression (summarized in **Figure 1**), but researchers have found that the energy demands of immune cells and tissues/organs are significantly increased (30). Aerobic glycolysis can rapidly generate energy to meet the high metabolic requirements of the cells (31). Inflammatory cytokines such as TNF-α, IL-1β, and IL-6 promote glycolysis by activating HIF-1α (Hypoxia-Inducible Factor 1-alpha), which in turn upregulates the expression of glycolysis-related genes, leading to increased glucose uptake and lactate production (32–34). Under physiological conditions, cells primarily rely on OXPHOS in the mitochondria to generate energy, a process that is highly efficient but dependent on the availability of oxygen. However, in the pathological context of sepsis, the energy metabolism of immune cells and organ cells undergoes significant reprogramming, shifting from OXPHOS to glycolysis, even when oxygen supply is sufficient (35, 36).

This metabolic shift is likely an adaptive response to meet the rapidly increased energy demands during the inflammatory response. Although glycolysis is less efficient in terms of energy production, it can rapidly synthesize ATP under hypoxic or low-oxygen conditions and also generates various metabolic intermediates that support biosynthetic activities within the cell, such as cell proliferation, differentiation, and the synthesis of inflammatory cytokines (37).

In the early stages of the inflammatory response, this metabolic reprogramming facilitates the rapid activation of immune cells and the timely initiation of the inflammatory response, playing an important physiological role. However, long-term reliance on glycolysis for energy production may lead to cellular dysfunction and immune suppression (38–40). This is because the energy output from glycolysis is relatively limited, and the sustained high levels of lactate may cause damage to cells and tissues.

### 2.2 Changes in metabolic products

#### 2.2.1 Lactate accumulation

In sepsis, the metabolic patterns of immune cells and tissue cells shift from OXPHOS to aerobic glycolysis (41). This shift is driven by inflammatory factors (such as TNF-α, IL-1β, and IL-6) through the activation of HIF-1α. HIF-1α further promotes the expression of glycolysis-related genes, increasing glucose uptake and lactate production. HIF-1 suppresses the TCA cycle by transactivating PDK1, which inactivates PDH to block pyruvate conversion to acetyl-CoA, and forced PDK1 expression in hypoxic HIF-1α-null cells increases ATP, reduces ROS, and prevents apoptosis (42). Additionally, mitochondrial dysfunction forces cells to rely more heavily on glycolysis for energy generation, further exacerbating lactate accumulation (43). Sepsis also causes mitochondrial damage, reducing the efficiency of OXPHOS (44). As cells become unable to effectively utilize pyruvate for OXPHOS, the pathway converting pyruvate to lactate becomes the primary mode of energy production.

Lactate accumulation, to some extent, helps maintain cellular metabolic homeostasis (45). Particularly during increased energy demands, lactate can serve as a rapid energy source, supporting normal cellular function (46). However, excessive lactate levels can lead to acidosis, impairing cellular function and tissue perfusion (47). Acidosis further suppresses mitochondrial function, worsening cellular energy metabolism disorders and creating a vicious cycle (48). High lactate levels may also contribute to immunosuppression by inhibiting the activation and function of immune cells. This not only impairs the body’s ability to clear pathogens but may also increase the risk of secondary infections (49, 50).

Lactate is not merely a metabolic byproduct; it also possesses immunomodulatory effects. It can suppress inflammatory responses and promote the formation of anti-inflammatory phenotypes in immune cells (such as M2 macrophages) (51). By activating receptors like GPR81, lactate inhibits the TLR signaling pathway and reduces the production of inflammatory cytokines, thereby partially mitigating inflammation (52). Lactate can serve as an energy substrate, supporting cellular metabolic needs during hypoxia or increased energy demand (53, 54). Furthermore, lactate can reduce excessive inflammatory activation by activating inhibitors of the NLRP3 inflammasome, protecting cells from inflammatory damage (55, 56). However, lactate accumulation may also impair cellular function and cause tissue damage by disrupting acid-base balance and energy metabolism (57, 58). Particularly in cardiac and brain tissues, lactate accumulation can lead to severe dysfunction, affecting organ performance (59, 60).

The accumulation of lactate in sepsis is a complex metabolic phenomenon with both beneficial and detrimental effects. Understanding the mechanisms of lactate’s role in sepsis is crucial for developing targeted therapeutic strategies. These may include modulating the glycolytic pathway or improving mitochondrial function to control lactate levels, thereby alleviating inflammation and tissue damage, and ultimately improving patient outcomes.

#### 2.2.2 Ketogenesis suppression

In sepsis, ketone body production may be suppressed (61, 62). Under normal conditions, ketone body generation primarily relies on FAO in the liver and the accumulation of acetyl-CoA (63). However, in septic patients, mitochondrial dysfunction and inhibition of the FAO pathway leads to reduced acetyl-CoA production, thereby impairing ketone body synthesis (64, 65). Furthermore, hyperglycemia and insulin resistance in sepsis may further reduce ketone body production by suppressing lipolysis and fatty acid release (66, 67).

Ketone bodies exhibit significant anti-inflammatory effects. Among them, β-hydroxybutyrate (BHB), a major ketone body, can suppress inflammatory responses through multiple mechanisms. BHB can activate the GPR109A receptor (68). Activation of this receptor inhibits NLRP3 inflammasome activation, thereby reducing the release of inflammatory cytokines (such as IL-1β and IL-18) (69). Overactivation of the NLRP3 inflammasome is associated with various inflammatory diseases, including the inflammatory response in sepsis (70, 71). Therefore, by inhibiting the NLRP3 inflammasome, BHB can effectively mitigate inflammation and protect tissues from inflammatory damage.

Ketone bodies also possess cytoprotective effects. In terms of energy metabolism, ketone bodies can serve as an alternative energy source, particularly when glucose utilization is limited (63, 72). Their utilization can alleviate mitochondrial oxidative stress and improve cellular energy status (73). Additionally, ketone bodies can reduce the generation of reactive oxygen species (ROS) by modulating intracellular redox status, protecting cells from oxidative damage (74, 75). The protective effects of ketone bodies are particularly important in neural and cardiac tissues. Neurons and cardiomyocytes have high energy demands, and a deficiency in ketone bodies may lead to energy metabolism disorders in these cells, potentially causing neural damage and cardiac dysfunction (76–78).

Ketone bodies play important anti-inflammatory and cytoprotective roles in sepsis. Although sepsis may lead to reduced ketone body production, supplementing ketone bodies or promoting their generation through other means could become an effective therapeutic strategy to mitigate inflammation and protect neural and cardiac tissues from damage. Future research needs to further explore the specific mechanisms of ketone bodies in sepsis and how modulating ketone body production can improve patient outcomes.

#### 2.2.3 Elevated free fatty acids

Plasma levels of free fatty acids (FFAs) are significantly elevated in septic patients (79, 80). This phenomenon results from several interrelated factors: 1)The stress response induced by sepsis activates the sympathetic nervous system and adrenal cortex, leading to the release of hormones such as catecholamines and cortisol. These hormones promote lipolysis in adipose tissue, releasing large amounts of FFAs into the bloodstream (81–83). 2)Septic patients often exhibit insulin resistance, impairing insulin’s ability to effectively suppress lipolysis, which further exacerbates FFA release (84). 3)During sepsis, the body’s energy demands increase significantly. Adipose tissue, as the primary energy reserve organ, releases FFAs derived from its breakdown into the blood to meet these demands (85, 86). 4)Despite significantly elevated FFA levels, their oxidation and utilization are impaired. Sepsis causes mitochondrial damage and reduces the efficiency of OXPHOS, preventing cells from effectively utilizing FFAs for oxidative metabolism. This leads to intracellular accumulation of FFAs (87). PPARα(peroxisome proliferators-activated receptor α), a key transcription factor regulating FAO, is significantly downregulated in sepsis. This reduces the expression of genes involved in FAO, further suppressing FFA utilization (88). Additionally, inflammatory cytokines such as TNF-α and IL-6 can inhibit PPARα activity, further hindering FFA oxidation (89, 90).

The excessive accumulation of FFAs leads to lipotoxicity, a cytotoxic state caused by dysregulated fatty acid metabolism. Excess FFAs accumulate within cells, exceeding the oxidative capacity of mitochondria, resulting in mitochondrial dysfunction. This mitochondrial dysfunction further exacerbates energy metabolism disorders, creating a vicious cycle. FFA accumulation can also induce endoplasmic reticulum stress, triggering the unfolded protein response (UPR). This dysregulates intracellular protein folding and degradation processes, further aggravating cellular damage. Lipotoxicity can induce apoptosis by activating caspase family proteases, leading to cell death. Apoptosis not only impairs individual cell function but can also contribute to organ dysfunction.

In summary, FFAs are significantly elevated in sepsis, but their oxidation is impaired, leading to intracellular accumulation and lipotoxicity. Lipotoxicity damages cells and tissues through multiple mechanisms, including mitochondrial dysfunction, endoplasmic reticulum stress, and apoptosis. This damage impairs not only individual cell function but can also lead to organ dysfunction, worsening the severity of sepsis. Therefore, modulating FFA metabolism and mitigating lipotoxicity may represent an effective therapeutic strategy to improve outcomes in septic patients. Future research needs to further elucidate the specific mechanisms of FFAs in sepsis and explore how modulating FFA metabolism can alleviate lipotoxicity to improve patient prognosis.

### 2.3 Alterations in metabolic signaling pathways

Sepsis is a complex systemic inflammatory response syndrome characterized by significant metabolic reprogramming during its onset and progression. These metabolic changes not only impact cellular energy metabolism but also profoundly influence immune responses and organ function. The following are key metabolic signaling pathways and their alterations in sepsis:

#### 2.3.1 HIF-1α pathway

HIF-1α, a key transcription factor under hypoxic conditions, primarily regulates cellular adaptive responses to oxygen deprivation (91). In sepsis, the stability and activity of HIF-1α are significantly enhanced. This is mainly driven by inflammatory factors (e.g., TNF-α, IL-1β, and IL-6) activating HIF-1α through multiple signaling pathways (92). Additionally, decreased intracellular oxygen levels resulting from mitochondrial dysfunction promote HIF-1α stabilization (93).

HIF-1α activates the expression of glycolysis-related genes, such as GLUT1 (glucose transporter 1), HK (hexokinase), PFK-1 (phosphofructokinase-1), and LDHA (lactate dehydrogenase A), thereby promoting glycolysis and lactate production (94, 95). HIF-1α regulates genes involved in iron metabolism, affecting iron absorption, transport, and storage, which subsequently alters cellular redox status (96). HIF-1α exacerbates inflammation by activating the expression of inflammatory cytokines (97, 98).

#### 2.3.2 mTOR pathway

mTOR (Mammalian Target of Rapamycin) is a critical regulator of cell growth and metabolism (99). In sepsis, mTOR complex 1 (mTORC1) activity is markedly increased (100). This is primarily due to inflammatory factors activating mTORC1 via the PI3K-AKT-mTOR pathway (101). Hyperglycemia and insulin resistance may further activate mTORC1 by enhancing glucose uptake and utilization (102, 103).

Activated mTORC1 increases HIF-1α stability and activity, further promoting glycolysis and lactate accumulation (104, 105). Overactivation of mTORC1 suppresses autophagy, impairing the clearance of damaged organelles and proteins, thereby exacerbating cellular injury (106, 107). mTORC1 hyperactivation may cause immune cell dysfunction (e.g., excessive activation of T cells and macrophages), intensifying inflammation and tissue damage (108, 109).

#### 2.3.3 AMPK pathway

AMPK (AMP-activated Protein Kinase) serves as a cellular energy sensor, primarily regulating energy metabolism by monitoring intracellular energy status (AMP/ATP ratio). AMPK activation promotes FAO, autophagy, and mitochondrial biogenesis to maintain energy homeostasis (110). In sepsis, AMPK activity is suppressed (111).

Inhibition occurs via inflammatory factors blocking AMPK activation through multiple pathways. Hyperglycemia and insulin resistance further exacerbate this suppression (112). Reduced AMPK activity decreases FAO and autophagy, disrupting energy metabolism and worsening cellular damage (113). AMPK suppression diminishes its anti-inflammatory effects, leading to exacerbated inflammation and tissue injury (114). Impaired AMPK activity compromises mitochondrial biogenesis and function, aggravating cellular energy deficits (115).

Key metabolic signaling pathways altered in sepsis include the HIF-1α, mTOR, and AMPK pathways. These changes profoundly impact cellular energy metabolism, immune responses, and organ function: 1) HIF-1α activation promotes glycolysis and inflammation. 2) mTOR hyperactivation causes immune dysfunction and tissue damage. 3)AMPK suppression disrupts energy balance and exacerbates inflammation. Understanding these mechanisms is essential for developing targeted therapies. Strategies such as modulating HIF-1α, mTOR, and AMPK activity may improve cellular metabolism and immune function, thereby reducing inflammation and tissue injury to enhance patient outcomes.

### 2.4 Alterations in metabolic enzymes

Sepsis is a systemic inflammatory response syndrome whose onset and progression are accompanied by significant metabolic reprogramming. Changes in the activity of metabolic enzymes are a crucial component of this reprogramming. These alterations not only impact cellular energy metabolism but also profoundly influence immune responses and the function of tissues and organs. The following details the changes in key metabolic enzymes during sepsis and their specific effects, as summarized in **Table 1**:

**Table 1:** Changes in key metabolic enzymes in sepsis.

Metabolic enzyme	Activity change	Impact on cellular metabolism and function
Pyruvate Dehydrogenase Complex (PDC)	Reduced activity	Decreased conversion of pyruvate to acetyl-CoA, impaired mitochondrial function, enhanced glycolysis
Phosphofructokinase-1 (PFK-1)	Increased activity	Enhanced glycolysis, accumulation of lactate
Acetyl-CoA Carboxylase (ACC)	Increased activity	Increased fatty acid synthesis, leading to lipotoxicity

#### 2.4.1 Pyruvate dehydrogenase complex

The pyruvate dehydrogenase complex (PDC) is a key enzyme responsible for converting pyruvate to acetyl-CoA (Acetyl-CoA), a process bridging glycolysis and the TCA cycle. The activity of PDC determines the efficiency of pyruvate entry into mitochondria for oxidative metabolism (116). In sepsis, PDC activity is significantly decreased (117). This phenomenon is primarily caused by the following factors: 1) Inflammatory cytokines such as TNF-α and IL-6 can inhibit PDC activity through multiple signaling pathways. For instance, these cytokines can activate protein kinases, leading to the phosphorylation and inactivation of PDC (118, 119). 2) Sepsis causes mitochondrial dysfunction, reducing the efficiency of OXPHOS and lowering intracellular oxygen concentration, which further suppresses PDC activity (120). 3) High lactate concentrations and an increased NADH/NAD+ ratio can feedback-inhibit PDC activity, reducing the amount of pyruvate entering the mitochondria (121, 122).

Reduced PDC activity impedes the conversion of pyruvate to acetyl-CoA, leading to intracellular pyruvate accumulation. This further promotes glycolysis and increases lactate production (123, 124). The inability of pyruvate to efficiently enter mitochondria for oxidative metabolism results in cellular energy metabolism disorders. Cells become dependent on ATP generated by glycolysis, which is less efficient and insufficient to meet high energy demands (125, 126). Blocked pyruvate entry into mitochondria reduces acetyl-CoA within the mitochondria, obstructing the TCA cycle and further exacerbating mitochondrial dysfunction (127).

#### 2.4.2 Phosphofructokinase-1

Phosphofructokinase-1 (PFK-1) is the key rate-limiting enzyme of glycolysis, responsible for converting fructose-6-phosphate to fructose-1, 6-bisphosphate. This step is the rate-limiting step of glycolysis (128, 129). In sepsis, PFK-1 activity is significantly increased (130). This phenomenon is primarily caused by the following factors: 1) HIF-1α, a key transcription factor under hypoxic conditions, exhibits significantly increased stability and activity in sepsis. HIF-1α can activate the expression of PFK-1, promoting glycolysis (95, 131). 2) Inflammatory cytokines can activate PFK-1 activity through various signaling pathways, further promoting glycolysis (132, 133).

Increased PFK-1 activity promotes glycolysis, enhancing glucose uptake and lactate production, thereby providing a rapid energy source for the cell. Enhanced glycolysis leads to increased lactate production (134, 135). Lactate accumulation may further inhibit mitochondrial function, exacerbating energy metabolism disorders. Lactate, as a metabolic product, can further exacerbate the inflammatory response by activating receptors such as TLR4 (58, 136).

#### 2.4.3 Acetyl-CoA carboxylase

Acetyl-CoA carboxylase (ACC) is a key enzyme in fatty acid synthesis, responsible for converting acetyl-CoA to malonyl-CoA (Malonyl-CoA). This process is the rate-limiting step of fatty acid synthesis (137, 138). In sepsis, ACC activity is significantly increased (139, 140). This phenomenon is primarily caused by the following factors: 1) Septic patients often exhibit insulin resistance, impairing insulin’s ability to effectively inhibit lipogenesis, which further activates ACC activity (141–143). 2) Inflammatory cytokines such as TNF-α and IL-6 can activate ACC activity, promoting fatty acid synthesis (118, 144).

Increased ACC activity promotes fatty acid synthesis, leading to intracellular fatty acid accumulation and exacerbating lipotoxicity (145). Fatty acid accumulation causes lipotoxicity, inducing mitochondrial dysfunction, endoplasmic reticulum stress, and apoptosis, further aggravating cellular damage (146). Increased fatty acid synthesis leads to intracellular energy metabolism imbalance, further worsening energy metabolism disorders.

Key alterations in metabolic enzymes during sepsis include the pyruvate dehydrogenase complex (PDC), phosphofructokinase-1 (PFK-1), and acetyl-CoA carboxylase (ACC), as summarized in **Table 2**. Changes in the activity of these enzymes not only impact cellular energy metabolism but also profoundly influence immune responses and the function of tissues and organs. Decreased PDC activity leads to pyruvate accumulation and mitochondrial dysfunction; increased PFK-1 activity promotes glycolysis and lactate accumulation; and increased ACC activity promotes fatty acid synthesis and lipotoxicity. Understanding the specific mechanisms of these metabolic enzymes in sepsis is crucial for developing targeted therapeutic strategies. Approaches such as modulating the activity of PDC, PFK-1, and ACC may improve cellular energy metabolism and immune responses, thereby alleviating inflammation and tissue damage, and ultimately improving patient outcomes.

**Table 2:** T cell metabolic reprogramming in sepsis.

Metabolic pathway	Change	Impact on T cell function
Oxidative Phosphorylation (OXPHOS)	Suppressed	Impaired T cell energy metabolism, reliance on less efficient glycolysis
Glycolysis	Enhanced	Accumulation of lactate, leading to acidosis and cellular dysfunction
Amino Acid Metabolism	Altered (e.g., activation of the tryptophan-kynurenine pathway)	Inhibition of T cell proliferation and function, increase in immunosuppressive cells (e.g., regulatory T cells)

## 3 Metabolism reprogramming shapes T cell function in sepsis

T lymphocytes, central orchestrators of the adaptive immune response, are severely compromised during sepsis, often falling into a state of dysfunction termed “T cell exhaustion” (147). As the core of adaptive immunity, the functional status of T cells directly determines the efficacy and durability of immune responses. Metabolic reprogramming within T cells is not just a consequence of sepsis but a central mechanism driving this immunosuppressive phenotype. This section focuses on the core mechanisms of T cell metabolic reprogramming in sepsis. By examining alterations in key pathways such as glycolysis, mitochondrial oxidative phosphorylation, and amino acid metabolism, it elucidates how these metabolic dysregulations directly lead to T cell dysfunction and consequently impair the efficacy of immune responses.

### 3.1 Sepsis-induced T cell exhaustion: core pathophysiological mechanisms

**Figure 2:**
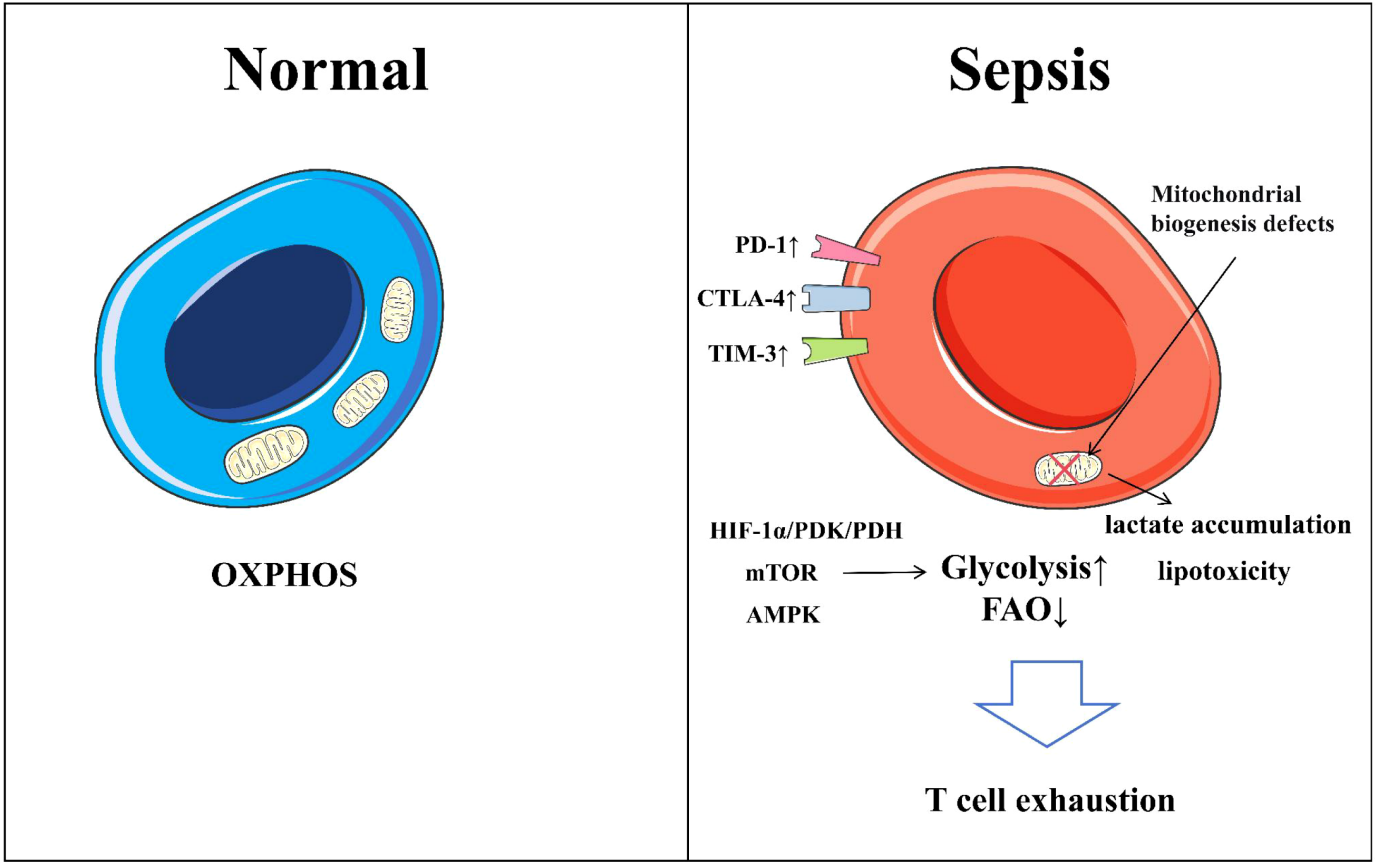
Alteration of T cell in sepsis. In sepsis, the metabolic pathways of T cells undergo significant changes, primarily characterized by a shift from oxidative phosphorylation (OXPHOS) to glycolysis. The activation of the mTOR signaling pathway and the suppression of the AMPK signaling pathway drive these metabolic alterations in T cells. The increased expression of inhibitory receptors on the surface of T cells (such as PD-1, CTLA-4, TIM-3, and LAG-3) further promotes T cell apoptosis and functional exhaustion.

T cells in sepsis patients exhibit typical characteristics of exhaustion (148), a unique state of cellular dysfunction centered on the progressive loss of effector functions, such as decreased proliferative capacity, reduced production of key cytokines (e.g., IL-2, IFN-γ), and diminished cytotoxicity. This exhausted state is driven by persistent antigen exposure and sustained negative signaling through inhibitory receptors, namely immune checkpoints (149) (**Figure 2**).

### 3.2 Metabolic reprogramming: the engine of T cell dysfunction in sepsis

In the complex clinical syndrome of sepsis, dysregulated host responses to infection lead to life-threatening organ dysfunction, with profound disturbances in the immune system being a core pathophysiological feature. Among these, T lymphocyte dysfunction—often manifested as “exhaustion” or immune paralysis—is a key factor contributing to increased susceptibility to secondary infections and elevated mortality.

Cellular metabolism is not merely a “power plant” providing energy (ATP) for cellular activities; it is a sophisticated signaling network regulating cell fate and function. For immune cells, changes in metabolic state directly dictate their entire lifecycle, from quiescence, activation, and effector function to memory formation. In healthy individuals, T cells can flexibly switch metabolic modes to meet diverse immune challenges (150, 151). However, within the persistent, catastrophic inflammatory storm of sepsis and the subsequent immunosuppressive environment, the intricate metabolic machinery within T cells suffers severe and prolonged disruption, plunging them into an “energy crisis”. This crisis is not simple energy shortage but a profound dysregulation of metabolic programming, forming the biochemical basis of the T cell exhaustion phenotype and ultimately leading to the collapse of the body’s immune defenses (152). As summarized in **Table 2**.

#### 3.2.1 Pathological glycolytic shift: the dysregulation of a double-edged sword

Under physiological conditions, T cell activation is accompanied by a significant metabolic shift: a rapid switch from the highly efficient mitochondrial OXPHOS mode characteristic of the resting state to a predominantly glycolytic mode for energy production (153). This phenomenon resembles the “Warburg effect” observed in tumor cells. Although less efficient in ATP yield, this shift rapidly provides essential macromolecule precursors (such as nucleotides, amino acids, and lipids) for cell division and the synthesis of effector molecules (like cytokines), which is crucial for supporting rapid immune responses (154).

However, in the pathological environment of sepsis, this normally transient adaptive shift becomes persistent and dominant, thereby acquiring pathological characteristics.

##### 3.2.1.1 Persistent glycolytic dependence

Studies have observed that CD4+ T cells surviving sepsis exhibit high dependence on glycolysis (155, 156). Unlike the benign process supporting proliferation in a healthy response, this metabolic program is “hijacked” and distorted within the chronic inflammatory microenvironment of sepsis. A core metabolic feature of terminally exhausted T cells is their reliance on glycolysis as the primary energy source, but this is typically accompanied by severely impaired mitochondrial function (157, 158). This “high sugar consumption, low output” metabolic state cannot effectively support long-term immune surveillance and effector functions, directly contributing to the immunosuppressive environment in the later stages of sepsis.

##### 3.2.1.2 Metabolic regulation by inhibitory signals

This pathological glycolytic state does not exist in isolation but is directly regulated by inhibitory signals within the immune microenvironment. PD-1 is a key marker of T cell exhaustion. Its binding to the ligand PD-L1 not only transmits inhibitory signals but also directly intervenes in cellular metabolism (159, 160). PD-1 signaling can effectively impair key pathways essential for activated T cell metabolism, such as the PI3K-Akt-mTOR pathway (161, 162). mTORC1 is a critical hub integrating nutrient and growth signals and is vital for promoting glycolysis and biosynthesis. Consequently, sustained PD-1 activation suppresses mTORC1 activity, thereby constraining T cell glycolytic potential and anabolic capacity (163). This renders the T cells unable to initiate the effective metabolic program necessary to support proliferation and cytotoxic functions even when encountering pathogens, trapping them in a state of functional paralysis.

#### 3.2.2 Widespread mitochondrial dysfunction: the core collapse of the energy system

If pathological glycolysis is a manifestation of T cell dysfunction, then widespread and persistent mitochondrial dysfunction is its fundamental intrinsic defect. This bioenergetic failure is a core mechanism driving the “exhaustion-like” phenotype of T cells in sepsis (164–166).

##### 3.2.2.1 Severe impairment of oxidative phosphorylation

Mitochondria are the sites where cells generate the vast majority of their ATP via OXPHOS. Studies consistently show that sepsis induces significant reductions in the protein expression and enzymatic activity of various complexes (particularly complexes I and IV) of the mitochondrial electron transport chain (ETC) in immune cells (167–169). This damage is closely related to the reduction or damage of mitochondrial DNA (mtDNA), as mtDNA encodes several key ETC subunits (170, 171). The loss of mtDNA directly prevents the proper assembly and function of the OXPHOS machinery, causing severe ATP deficiency (172). This forces cells to rely on inefficient glycolysis for mere survival, which is far insufficient to support complex immune functions such as cytokine production, proliferation, and the formation of effective immune memory.

##### 3.2.2.2 Decisive driver of T cell exhaustion

Maintaining robust and flexible mitochondrial function is key to distinguishing long-lived functional memory T cells from exhausted T cells. Functional memory T cells rely on mitochondrial FAO and OXPHOS to support their long-term survival and rapid recall responses upon reinfection (173–175). Within the progression of T cell exhaustion, there exists an intermediate stage: “progenitor exhausted T cells.” These cells retain some proliferative potential and partial effector function, and their survival depends precisely on mitochondrial FAO and OXPHOS (176, 177). However, in the persistently hostile microenvironment of sepsis, these cells gradually lose mitochondrial function, ultimately transforming into terminally exhausted T cells. Hallmarks of this transition include blocked mitochondrial biogenesis, decreased mitochondrial quality, and complete loss of FAO and OXPHOS capacity (44, 178). Therefore, mitochondrial dysfunction is not merely an accompanying phenomenon of T cell exhaustion but a core mechanism driving its occurrence.

#### 3.2.3 Dysregulation of key amino acid pathways: deterioration of the nutritional environment

Beyond the core disruptions in glucose and mitochondrial energy metabolism, sepsis also severely alters the metabolic environment of key amino acids, creating another barrier to T cell survival and function.

##### 3.2.3.1 Activation of the tryptophan-kynurenine pathway

During sepsis, inflammatory cytokines (such as IFN-γ) strongly induce high expression of an enzyme called Indoleamine 2, 3-dioxygenase 1 (IDO1) in various cells (179). IDO1 is a rate-limiting enzyme that catabolizes the essential amino acid tryptophan into kynurenine and a series of downstream metabolites (180). This process exerts dual immunosuppressive effects: first, it depletes tryptophan—essential for T cell proliferation—in the local microenvironment, creating “nutrient starvation” that directly inhibits T cell growth; second, the produced kynurenine itself is a potent immunosuppressive molecule that actively suppresses effector T cell activity and promotes the generation and differentiation of immunosuppressive Tregs (181–183). Arginine is another conditionally essential amino acid crucial for T cell function (184). Furthermore, research indicates that alterations in tryptophan metabolism are directly linked to the glycolytic capacity and suppressive function remodeling of CD4+ Treg cells, revealing how amino acid metabolism synergizes with glucose metabolism to exacerbate immunosuppression (185).

##### 3.2.3.2 Depletion of the arginine-citrulline axis

Arginine is another conditionally essential amino acid crucial for T cell function. It is a precursor for T cell proliferation, differentiation, and the production of NO for cytotoxic functions (186). However, in the septic state, MDSCs and M2 macrophages express high levels of arginase, which breaks down arginine into ornithine and urea (187). This leads to severe depletion of local and systemic arginine, creating an “arginine desert” that effectively “starves” T cells, inhibiting their activation and proliferation. Clinically, direct arginine supplementation has limited efficacy due to the hepatic first-pass effect and rapid degradation by high levels of endogenous arginase. A more refined strategy is citrulline supplementation. Citrulline can be efficiently converted to arginine within the body and bypasses hepatic first-pass metabolism and arginase degradation, thereby effectively increasing plasma arginine levels and offering a potential avenue for restoring T cell function. Clinical studies also suggest that combined supplementation with glutamine and arginine can significantly reduce pro-inflammatory cytokine levels in sepsis patients, demonstrating potential for immune modulation and tissue repair (188).

This section elaborates on the central role of metabolic reprogramming in driving T cell exhaustion during sepsis. T cell dysfunction is not merely a passive consequence but is actively driven by fundamental alterations in their metabolic state. These changes include a pathological shift from efficient oxidative phosphorylation to persistent and inefficient glycolysis, severe mitochondrial dysfunction (e.g., impaired electron transport chain, reduced mtDNA), and the depletion or inhibition of key amino acid metabolism pathways (such as tryptophan and arginine). Collectively, these metabolic defects deprive T cells of the energy and biosynthetic precursors essential for effector functions, sustained proliferation, and the formation of immunological memory, thereby actively driving and sustaining their “exhausted” state. This research traces the root of T cell dysfunction back to the cellular metabolic level, revealing a deeper mechanism of immunosuppression. Future studies should utilize technologies like single-cell multi-omics to precisely map the dynamic metabolic profiles of different T cell subsets throughout the course of sepsis. A key focus should be deciphering how metabolites solidify the exhaustion program by regulating epigenetics (e.g., histone modifications, DNA methylation). Furthermore, exploring the roles of metabolism beyond glucose, such as lipid and nucleotide metabolism, in T cell exhaustion will be crucial for discovering novel therapeutic targets.

## 4 Therapeutic strategies targeting metabolic pathways to restore T cell function in sepsis

**Figure 3:**
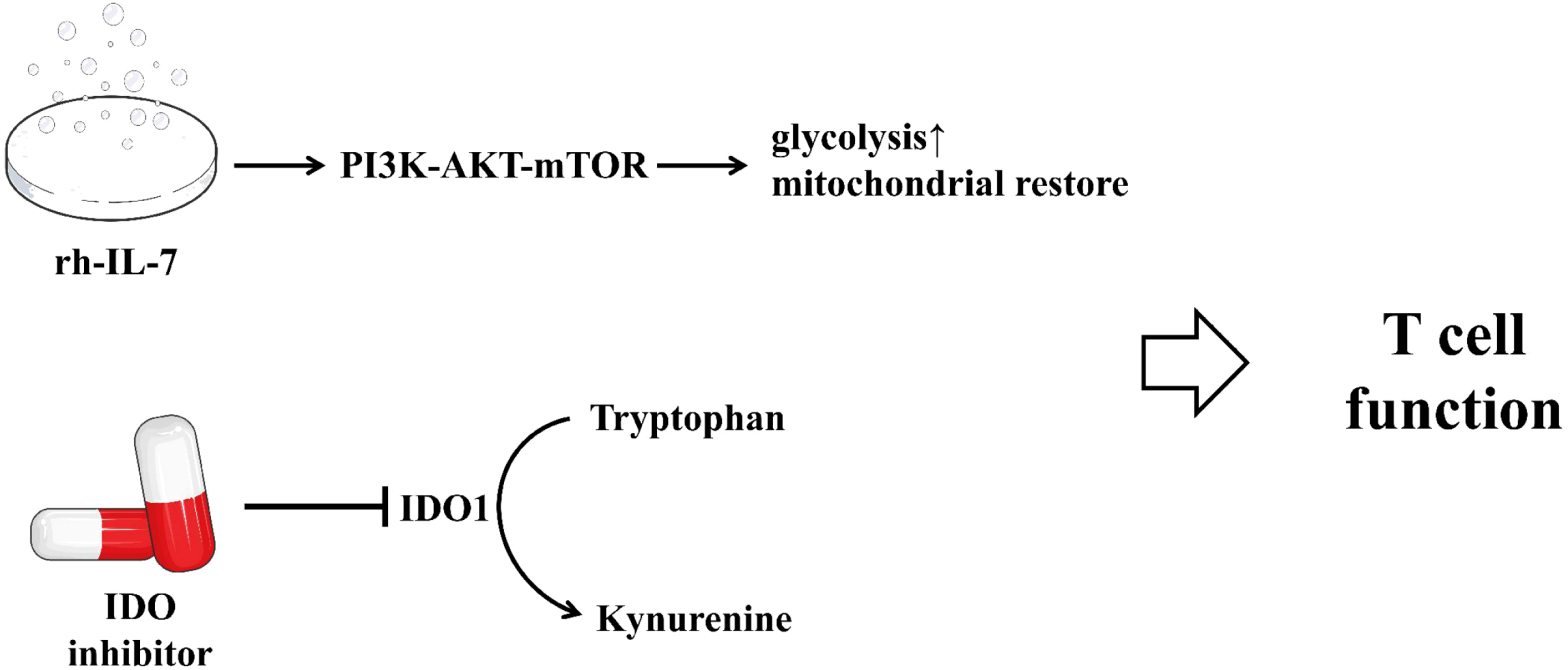
Potential therapeutic strategies targeting metabolic pathways to restore T cell function in sepsis. Recombinant human interleukin-7(rh-IL-7) enhances T-cell glycolysis and oxidative phosphorylation by activating the mTOR signaling pathway, thereby promoting T-cell proliferation and functional recovery. It increases the number of CD4^+^ and CD8^+^T cells in the peripheral blood of sepsis patients, improving immune function.IDO1 inhibitors reduce kynurenine production and increase tryptophan levels by inhibiting IDO1 enzyme activity, helping to restore T-cell function and alleviate immune suppression. However, further research is needed to explore their application in sepsis.

Sepsis is a life-threatening organ dysfunction caused by a dysregulated host response to infection. A core feature of its pathophysiology is progressive immunosuppression, in which T-cell exhaustion plays a pivotal role. Recent research has profoundly revealed that this immune cell dysfunction is not an isolated event but is driven by underlying cellular metabolic derangements. The metabolic characteristics of the exhausted T cell state, such as impaired glycolysis and mitochondrial OXPHOS capacity, render T cells incapable of effective proliferation, differentiation, and execution of effector functions. Recognizing this metabolic failure as the core mechanism driving T-cell exhaustion opens novel avenues for therapeutic intervention in sepsis. “Immunometabolism” therapies have emerged, with the central goal of restoring the fighting capacity of immune cells by correcting the underlying metabolic defects, thereby breaking the vicious cycle of sepsis-induced immunosuppression. The following summarizes the application of some potential therapeutic targets in sepsis. The specific mechanisms can be referred to in **Figure 3**.

### 4.1 Restoring T cell proliferation and function via recombinant human interleukin-7

Recombinant human IL-7 (rh-IL-7), such as the drug CYT107, represents a highly promising immunoadjuvant therapy aimed directly at countering sepsis-induced severe lymphopenia. The efficacy of rh-IL-7 is fundamentally rooted in its powerful immunometabolic remodeling capabilities (189, 190).

#### 4.1.1 Deep dive into mechanism of action: from mTOR activation to mitochondrial function remodeling

The mechanism of IL-7 action extends far beyond simple cytokine stimulation; it systematically repairs the metabolic engine of exhausted T cells by activating key intracellular signaling pathways.

##### 4.1.1.1 Activation of the mTOR pathway

In sepsis, IL-7 levels in patients decrease, and the expression of its receptor (IL-7R) on the T cell surface is significantly reduced, collectively leading to diminished activity of downstream signaling pathways (191, 192). Administration of rh-IL-7 effectively compensates for this defect. Studies confirm that IL-7 therapy strongly activates the mammalian target of rapamycin (mTOR) pathway, specifically mTORC1, in T lymphocytes. mTOR is a central regulatory hub for cell metabolism, growth, proliferation, and survival (99). IL-7 activates upstream AKT and STAT5 signaling pathways, converging on mTORC1 to initiate a series of metabolic reprogramming events (193). Experimental evidence shows that the beneficial effects of IL-7 are completely blocked by the mTOR inhibitor rapamycin, directly proving the core role of the mTOR pathway in this process (194).

##### 4.1.1.2 Remodeling glucose metabolism

Activated mTORC1 immediately upregulates the expression of key metabolic proteins, most notably Glucose Transporter 1 (GLUT1) (195). Sepsis T cells exhibit reduced GLUT1 expression, leading to severely impaired glucose uptake capacity, unable to meet the substantial energy demands for activation and proliferation. IL-7-mediated mTOR activation significantly increases GLUT1 density on the T cell surface, thereby dramatically boosting glucose uptake and providing ample “fuel” for cell proliferation. This metabolic “rescue” is a prerequisite for restoring T cell proliferative capacity (193, 196).

##### 4.1.1.3 Repairing mitochondrial function

Restoring OXPHOS: Sepsis not only suppresses glycolysis but also causes severe mitochondrial dysfunction, manifested as decreased mitochondrial membrane potential, impaired respiratory function, and inefficient ATP production (44). mTORC1 not only regulates glycolysis but is also a key factor in maintaining normal mitochondrial function and promoting OXPHOS. mTORC1 activity positively correlates with mitochondrial membrane potential, maximal respiratory capacity, and intracellular ATP content. Therefore, by activating mTORC1, IL-7 not only restores glycolysis but may also directly or indirectly repair damaged mitochondria, improving OXPHOS capacity (197). Although no published studies currently provide specific quantitative data on changes in parameters like CD8+ T cell mitochondrial basal respiration or ATP-linked oxygen consumption rates in septic patients before and after IL-7 treatment, based on the known functions of mTOR and evidence that IL-7 improves T cell basal metabolic status, it is reasonable to infer that IL-7 therapy can correct the metabolic block preventing septic T cells from shifting from inefficient glycolysis to more efficient OXPHOS. Theoretically, this could be precisely measured using platforms like Seahorse XF.

#### 4.1.2 Clinical evidence

The IRIS-7 series of clinical trials (primarily NCT02640807 and NCT02797431) represents a landmark study evaluating the application of rh-IL-7 (CYT107) in sepsis (198). It was a randomized, double-blind, placebo-controlled phase III trial conducted across multiple academic centers in France and the US, specifically enrolling critically ill patients with septic shock and severe lymphopenia. According to released trial information, IRIS-7 successfully met its primary endpoints. Results indicated that CYT107 administration was safe and well-tolerated, with no observed signs of induced cytokine storms or exacerbated inflammatory responses. Regarding efficacy, CYT107 demonstrated potent immune reconstitution capability: treated patients experienced a greater than threefold increase in circulating CD4+ and CD8+ T cell counts, and this significant lymphocyte rebound effect persisted for several weeks after treatment cessation. This robustly proves the effectiveness of rh-IL-7 in reversing the core pathological feature of sepsis—lymphopenia.

### 4.2 Modulating the kynurenine pathway via IDO1 inhibitors

Unlike IL-7’s strategy of directly “refueling” T cells, IDO1 inhibitors aim to restore T cell function by “lifting the blockade”. The IDO1-Kynurenine pathway is a major endogenous immunosuppressive mechanism that is aberrantly activated in sepsis.

#### 4.2.1 Mechanism of action: releasing the dual shackles of tryptophan deprivation and kynurenine toxicity

##### 4.2.1.1 Aberrant activation of IDO1 in sepsis

IDO1 is a key rate-limiting enzyme that catalyzes the breakdown of the essential amino acid tryptophan into kynurenine and its downstream metabolites. During sepsis, inflammatory cytokines induce high expression of IDO1 in immune cells, particularly macrophages and dendritic cells. This leads to two major immunosuppressive consequences:

1) Tryptophan Depletion: T cell proliferation and function are highly dependent on tryptophan supply. Overactivation of IDO1 depletes tryptophan in the microenvironment, akin to cutting off the T cells’ “food supply”, plunging them into a state of “starvation” (182).

2) Kynurenine Accumulation: The generated kynurenine and its metabolites are not inactive byproducts; they are potent immunosuppressive molecules themselves. Kynurenine can directly induce T cell apoptosis, inhibit T cell proliferation, and promote the differentiation of immunosuppressive Tregs (199).

##### 4.2.1.2 The “detoxifying” effect of IDO1 inhibitors

IDO1 inhibitors, such as the extensively studied Epacadostat and BMS-986205 (Linrodostat), work by specifically binding to and inhibiting the activity of the IDO1 enzyme, directly blocking the conversion of tryptophan to kynurenine (200–202). Theoretically, this offers dual benefits:

1. Restoring Tryptophan Levels: Ensuring sufficient tryptophan for T cell protein synthesis and activation signaling.
2. Reducing Kynurenine Levels: Relieving the direct toxicity and inhibitory effects of Kyn on T cells, thereby restoring T cell proliferative capacity and effector function, and enhancing pathogen clearance.

#### 4.2.2 Current state of preclinical and clinical evidence and the significant knowledge gap

Despite the highly attractive mechanism of IDO1 inhibitors, research on their application in sepsis lags significantly behind that in cancer immunotherapy.

##### 4.2.2.1 Evidence from oncology

Currently, most preclinical and clinical data on Epacadostat and BMS-986205 come from oncology. In cancer clinical trials, these inhibitors have demonstrated clear pharmacodynamic effects. For instance, BMS-986205 has been shown to significantly reduce plasma and tumor tissue kynurenine levels in cancer patients by up to 90%, effectively lowering the Kyn/Trp ratio (203). This improvement in biochemical markers is accompanied by enhanced T cell proliferation and infiltration within tumors. These data provide strong proof-of-concept for the ability of these drugs to target IDO1 and reverse immunosuppression.

##### 4.2.2.2 Knowledge gap in sepsis

However, directly extrapolating these findings to sepsis requires extreme caution. To date, there are almost no publicly available clinical trial data specific to sepsis, and there is a lack of published literature using modern, potent IDO1 inhibitors like Epacadostat or BMS-986205 in relevant animal models of sepsis. To address this gap, future preclinical studies are crucial. An ideal study design would employ clinically relevant sepsis models, such as the cecal ligation and puncture (CLP) murine model. In this model, researchers would administer inhibitors like Epacadostat and perform quantitative measurements at different time points. Key assessment metrics should include: 1) Pathogen Clearance Kinetics: Directly evaluating whether the drug enhances the host’s bacterial clearance capacity by quantifying bacterial colony-forming units in peritoneal lavage fluid and blood. 2) Restoration of T Cell Metabolic Function: Directly measuring glycolytic flux (e.g., extracellular acidification rate, ECAR) in CD8+ T cells isolated from septic mice using flow cytometry or Seahorse cellular energy metabolism analysis to validate if IDO1 inhibition indeed restores T cell metabolic activity.Until these critical preclinical data are obtained, the potential of IDO1 inhibitors for sepsis treatment remains largely theoretical.

This section systematically reviews therapeutic strategies aimed at restoring T cell function in sepsis by targeting metabolic pathways. The core concept is to directly correct the metabolic defects in T cells through “immunometabolic” interventions. For instance, recombinant human IL-7, by activating the mTOR signaling pathway, synergistically enhances T cell glucose uptake, glycolysis, and oxidative phosphorylation capacity, thereby effectively promoting their *in vivo* expansion and survival. Conversely, IDO1 inhibitors work by blocking the tryptophan-kynurenine pathway, lifting the “dual shackles” on T cells (tryptophan depletion and kynurenine toxicity) to improve the immune microenvironment and restore T cell function. These strategies represent a paradigm shift from non-specific immunosuppression towards precise immunometabolic reconstitution. Future research directions should focus on systematically evaluating the synergistic effects of these metabolic interventions with existing therapies (such as immune checkpoint inhibitors and antimicrobial agents) to design rational combination or sequential treatment regimens. Concurrently, in-depth preclinical studies are urgently needed to validate new targets (e.g., the efficacy of IDO1 inhibitors in sepsis models) and to promote the development of biomarkers capable of monitoring the metabolic status of immune cells in patients in real-time, ultimately paving the way for personalized immunotherapy in sepsis.

## 5 Conclusion

Sepsis-induced immunosuppression and the associated T cell exhaustion have emerged as decisive factors determining long-term patient outcomes. This review has systematically elaborated the central role of metabolic reprogramming in driving T cell dysfunction—it is not merely a consequence of immune paralysis but acts as a fundamental driving mechanism that actively maintains the exhausted phenotype by altering energy metabolism, signal transduction, and epigenetic states. The collective metabolic defects, including persistent glycolytic dependency, severe mitochondrial dysfunction, and dysregulated metabolism of critical amino acids, constitute the metabolic foundation of T cell functional failure.

Although interventional strategies targeting key metabolic nodes, such as rhIL-7 and IDO1 inhibitors, have demonstrated potential in reversing immunosuppression, their translation into clinical practice still faces significant challenges. Building upon the current limitations and knowledge gaps, future research should prioritize the following cutting-edge directions, moving beyond simple associations to establish causality. Leveraging single-cell multi-omics technologies to map the complete metabolic profiles of different T cell subsets across various stages of sepsis at single-cell resolution will be crucial for uncovering the causal relationships between metabolic reprogramming and cell fate decisions. A particular focus should be placed on deciphering the specific mechanisms of metabolism-epigenetics cross-talk, clarifying how specific metabolites solidify the T cell exhaustion program by regulating histone modifications and DNA methylation.

Furthermore, the exploration must extend beyond glucose metabolism to deeply investigate the roles of lipid metabolism and nucleotide metabolism in T cell exhaustion, thereby uncovering novel therapeutic targets. Systematically evaluating the synergistic effects between metabolic interventions and existing therapies, such as immune checkpoint inhibitors and antimicrobial agents, is essential for designing rational sequential or combination treatment regimens to overcome the limitations of monotherapy. Concurrently, addressing the core challenges in translational medicine requires establishing non-invasive or minimally invasive biomarkers capable of monitoring the metabolic status of immune cells in patients in real-time. This will provide the tools for precise “metabolic phenotyping” and patient stratification. It is equally critical to define the critical time windows for metabolic interventions and elucidate the timing dependency and potential risks of metabolic modulation during different pathological stages of sepsis. Developing next-generation animal models that better mimic the immunometabolic features of human sepsis will enhance the predictive value of preclinical research.

Expanding the systems biology perspective is also imperative. Future work should investigate the metabolic-level interactions between T cells and other components like myeloid cells and stromal cells, situating T cell metabolism within the broader metabolic ecosystem of the sepsis microenvironment. Exploring inter-organ metabolic crosstalk and its distal regulatory mechanisms on systemic immune cell function represents another promising frontier. Finally, promoting methodological innovation and standardization is key. Efforts should focus on standardizing immunometabolic detection technologies and establishing unified analytical workflows to ensure comparability across studies. Integrating computational biology and artificial intelligence to construct virtual T cell models capable of predicting the outcomes of metabolic interventions will guide the development of personalized treatment strategies.

In summary, targeting T cell metabolism represents a paradigm shift in sepsis treatment, moving from “non-specific immunosuppression” towards “precision immune reconstitution.” By addressing these challenges systematically through interdisciplinary collaboration, we hold the promise of ultimately overcoming the dilemma of sepsis-induced immune paralysis and delivering breakthrough solutions to this long-standing critical challenge in medicine.

## References

[1] van der Poll T Shankar-Hari M Wiersinga WJ . The immunology of sepsis. Immunity. (2021) 54:2450–64. doi: 10.1016/j.immuni.2021.10.012, PMID: 34758337

[2] Jarczak D Kluge S Nierhaus A . Sepsis—Pathophysiology and therapeutic concepts. Front Med (Lausanne). (2021) 8:628302. doi: 10.3389/fmed.2021.628302, PMID: 34055825 PMC8160230

[3] Singer M Deutschman CS Seymour CW Shankar-Hari M Annane D Bauer M . The third international consensus definitions for sepsis and septic shock (Sepsis-3). JAMA. (2016) 315:801–10. doi: 10.1001/jama.2016.0287, PMID: 26903338 PMC4968574

[4] Markwart R Saito H Harder T Tomczyk S Cassini A Fleischmann-Struzek C . Epidemiology and burden of sepsis acquired in hospitals and intensive care units: a systematic review and meta-analysis. Intensive Care Med. (2020) 46:1536–51. doi: 10.1007/s00134-020-06106-2, PMID: 32591853 PMC7381455

[5] Rudd KE Johnson SC Agesa KM Shackelford KA Tsoi D Kievlan DR . Global, regional, and national sepsis incidence and mortality, 1990–2017: analysis for the Global Burden of Disease Study. Lancet. (2020) 395:200–11. doi: 10.1016/S0140-6736(19)32989-7, PMID: 31954465 PMC6970225

[6] Lei S Li X Zhao H Xie Y Li J . Prevalence of sepsis among adults in China: A systematic review and meta-analysis. Front Public Health. (2022) 10:977094. doi: 10.3389/fpubh.2022.977094, PMID: 36304237 PMC9596150

[7] Liu Y-C Yao Y Yu M-M Gao Y-L Qi A-L Jiang T-Y . Frequency and mortality of sepsis and septic shock in China: a systematic review and meta-analysis. BMC Infect Dis. (2022) 22:564. doi: 10.1186/s12879-022-07543-8, PMID: 35729526 PMC9210671

[8] Delano MJ Ward PA . The immune system’s role in sepsis progression, resolution, and long-term outcome. Immunol Rev. (2016) 274:330–53. doi: 10.1111/imr.12499, PMID: 27782333 PMC5111634

[9] Williams Roberson S Nwosu S Collar EM Kiehl AL Harrison FE Bastarache J . Association of vitamin C, thiamine, and hydrocortisone infusion with long-term cognitive, psychological, and functional outcomes in sepsis survivors: A secondary analysis of the vitamin C, thiamine, and steroids in sepsis randomized clinical trial. JAMA Netw Open. (2023) 6:e230380. doi: 10.1001/jamanetworkopen.2023.0380, PMID: 36853612 PMC9975932

[10] Seymour CW Gesten F Prescott HC Friedrich ME Iwashyna TJ Phillips GS . Time to treatment and mortality during mandated emergency care for sepsis. N Engl J Med. (2017) 376:2235–44. doi: 10.1056/NEJMoa1703058, PMID: 28528569 PMC5538258

[11] Zampieri FG Bagshaw SM Semler MW . Fluid therapy for critically ill adults with sepsis: A review. JAMA. (2023) 329:1967–80. doi: 10.1001/jama.2023.7560, PMID: 37314271

[12] Prescott HC Angus DC . Enhancing recovery from sepsis: A review. JAMA. (2018) 319:62–75. doi: 10.1001/jama.2017.17687, PMID: 29297082 PMC5839473

[13] Tang F Yuan H Li X Qiao L . Effect of delayed antibiotic use on mortality outcomes in patients with sepsis or septic shock: A systematic review and *meta*-analysis. Int Immunopharmacol. (2024) 129:111616. doi: 10.1016/j.intimp.2024.111616, PMID: 38310764

[14] Ahmed SK Hussein S Qurbani K Ibrahim RH Fareeq A Mahmood KA . Antimicrobial resistance: Impacts, challenges, and future prospects. J Med Surgery Public Health. (2024) 2:100081. doi: 10.1016/j.glmedi.2024.100081

[15] Rousseau A-F Prescott HC Brett SJ Weiss B Azoulay E Creteur J . Long-term outcomes after critical illness: recent insights. Crit Care. (2021) 25:108. doi: 10.1186/s13054-021-03535-3, PMID: 33731201 PMC7968190

[16] Wang T Derhovanessian A De Cruz S Belperio JA Deng JC Hoo GS . Subsequent infections in survivors of sepsis: epidemiology and outcomes. J Intensive Care Med. (2014) 29:87–95. doi: 10.1177/0885066612467162, PMID: 23753224 PMC4393330

[17] Gao X Cai S Li X Wu G . Sepsis-induced immunosuppression: mechanisms, biomarkers and immunotherapy. Front Immunol. (2025) 16:1577105. doi: 10.3389/fimmu.2025.1577105, PMID: 40364841 PMC12069044

[18] Delano MJ Ward PA . Sepsis-induced immune dysfunction: can immune therapies reduce mortality? J Clin Invest. (2016) 126:23–31. doi: 10.1172/JCI82224, PMID: 26727230 PMC4701539

[19] Willmann K Moita LF . Physiologic disruption and metabolic reprogramming in infection and sepsis. Cell Metab. (2024) 36:927–46. doi: 10.1016/j.cmet.2024.02.013, PMID: 38513649

[20] Van Wyngene L Vandewalle J Libert C . Reprogramming of basic metabolic pathways in microbial sepsis: therapeutic targets at last? EMBO Mol Med. (2018) 10:e8712. doi: 10.15252/emmm.201708712, PMID: 29976786 PMC6079534

[21] Hirasawa H Oda S Nakamura M . Blood glucose control in patients with severe sepsis and septic shock. World J Gastroenterol. (2009) 15:4132–6. doi: 10.3748/wjg.15.4132, PMID: 19725146 PMC2738808

[22] Wernly B Lichtenauer M Hoppe UC Jung C . Hyperglycemia in septic patients: an essential stress survival response in all, a robust marker for risk stratification in some, to be messed with in none. J Thorac Dis. (2016) 8:E621–4. doi: 10.21037/jtd.2016.05.24, PMID: 27501420 PMC4958822

[23] Ming S Li X Xiao Q Qu S Wang Q Fang Q . TREM2 aggravates sepsis by inhibiting fatty acid oxidation via the SHP1/BTK axis. J Clin Invest. (2024) 135:e159400. doi: 10.1172/JCI159400, PMID: 39405126 PMC11684808

[24] Li R Li X Zhao J Meng F Yao C Bao E . Mitochondrial STAT3 exacerbates LPS-induced sepsis by driving CPT1a-mediated fatty acid oxidation. Theranostics. (2022) 12:976–98. doi: 10.7150/thno.63751, PMID: 34976224 PMC8692896

[25] Raman S Klein N Kwan A Hubank M Rahman S Rashid A . Oxidative phosphorylation gene expression falls at onset and throughout the development of meningococcal sepsis-induced multi-organ failure in children. Intensive Care Med. (2015) 41:1489–90. doi: 10.1007/s00134-015-3817-y, PMID: 25920543 PMC4502289

[26] Peng M Yin N Chhangawala S Xu K Leslie CS Li MO . Aerobic glycolysis promotes T helper 1 cell differentiation through an epigenetic mechanism. Science. (2016) 354:481–4. doi: 10.1126/science.aaf6284, PMID: 27708054 PMC5539971

[27] Menk AV Scharping NE Moreci RS Zeng X Guy C Salvatore S . Early TCR signaling induces rapid aerobic glycolysis enabling distinct acute T cell effector functions. Cell Rep. (2018) 22:1509–21. doi: 10.1016/j.celrep.2018.01.040, PMID: 29425506 PMC5973810

[28] Tannahill GM Curtis AM Adamik J Palsson-McDermott EM McGettrick AF Goel G . Succinate is an inflammatory signal that induces IL-1β through HIF-1α. Nature. (2013) 496:238–42. doi: 10.1038/nature11986, PMID: 23535595 PMC4031686

[29] Gu K Wu A Liu C Yu B He J Lai X . Absence of gut microbiota alleviates iron overload-induced colitis by modulating ferroptosis in mice. J Adv Res. (2024), S2090–1232(24)00608–8. doi: 10.1016/j.jare.2024.12.030, PMID: 39710300 PMC12793762

[30] Wiersinga WJ van der Poll T . Immunopathophysiology of human sepsis. EBioMedicine. (2022) 86:104363. doi: 10.1016/j.ebiom.2022.104363, PMID: 36470832 PMC9783164

[31] Lunt SY Vander Heiden MG . Aerobic glycolysis: meeting the metabolic requirements of cell proliferation. Annu Rev Cell Dev Biol. (2011) 27:441–64. doi: 10.1146/annurev-cellbio-092910-154237, PMID: 21985671

[32] Tan Q Huang Q Ma YL Mao K Yang G Luo P . Potential roles of IL-1 subfamily members in glycolysis in disease. Cytokine Growth Factor Rev. (2018) 44:18–27. doi: 10.1016/j.cytogfr.2018.11.001, PMID: 30470512

[33] Wu L Jin Y Zhao X Tang K Zhao Y Tong L . Tumor aerobic glycolysis confers immune evasion through modulating sensitivity to T cell-mediated bystander killing via TNF-α. Cell Metab. (2023) 35:1580–1596.e9. doi: 10.1016/j.cmet.2023.07.001, PMID: 37506695

[34] Xu S Deng K-Q Lu C Fu X Zhu Q Wan S . Interleukin-6 classic and trans-signaling utilize glucose metabolism reprogramming to achieve anti- or pro-inflammatory effects. Metabolism. (2024) 155:155832. doi: 10.1016/j.metabol.2024.155832, PMID: 38438106

[35] Liu J Zhou G Wang X Liu D . Metabolic reprogramming consequences of sepsis: adaptations and contradictions. Cell Mol Life Sci. (2022) 79:456. doi: 10.1007/s00018-022-04490-0, PMID: 35904600 PMC9336160

[36] Liu C Wei W Huang Y Fu P Zhang L Zhao Y . Metabolic reprogramming in septic acute kidney injury: pathogenesis and therapeutic implications. Metabolism. (2024) 158:155974. doi: 10.1016/j.metabol.2024.155974, PMID: 38996912

[37] Shen Y Dinh HV Cruz ER Chen Z Bartman CR Xiao T . Mitochondrial ATP generation is more proteome efficient than glycolysis. Nat Chem Biol. (2024) 20:1123–32. doi: 10.1038/s41589-024-01571-y, PMID: 38448734 PMC11925356

[38] Kelly B O’Neill LAJ . Metabolic reprogramming in macrophages and dendritic cells in innate immunity. Cell Res. (2015) 25:771–84. doi: 10.1038/cr.2015.68, PMID: 26045163 PMC4493277

[39] Falkenberg KD Rohlenova K Luo Y Carmeliet P . The metabolic engine of endothelial cells. Nat Metab. (2019) 1:937–46. doi: 10.1038/s42255-019-0117-9, PMID: 32694836

[40] Tufail M Jiang C-H Li N . Altered metabolism in cancer: insights into energy pathways and therapeutic targets. Mol Cancer. (2024) 23:203. doi: 10.1186/s12943-024-02119-3, PMID: 39294640 PMC11409553

[41] Wasyluk W Zwolak A . Metabolic alterations in sepsis. J Clin Med. (2021) 10:2412. doi: 10.3390/jcm10112412, PMID: 34072402 PMC8197843

[42] Kim J Tchernyshyov I Semenza GL Dang CV . HIF-1-mediated expression of pyruvate dehydrogenase kinase: a metabolic switch required for cellular adaptation to hypoxia. Cell Metab. (2006) 3:177–85. doi: 10.1016/j.cmet.2006.02.002, PMID: 16517405

[43] San-Millán I . The key role of mitochondrial function in health and disease. Antioxid (Basel). (2023) 12:782. doi: 10.3390/antiox12040782, PMID: 37107158 PMC10135185

[44] Nedel W Deutschendorf C Portela LVC . Sepsis-induced mitochondrial dysfunction: A narrative review. World J Crit Care Med. (2023) 12:139–52. doi: 10.5492/wjccm.v12.i3.139, PMID: 37397587 PMC10308342

[45] Lee WD Weilandt DR Liang L MacArthur MR Jaiswal N Ong O . Lactate homeostasis is maintained through regulation of glycolysis and lipolysis. Cell Metab. (2025) 37:758–771.e8. doi: 10.1016/j.cmet.2024.12.009, PMID: 39889702 PMC11926601

[46] Li X Yang Y Zhang B Lin X Fu X An Y . Lactate metabolism in human health and disease. Signal Transduct Target Ther. (2022) 7:305. doi: 10.1038/s41392-022-01151-3, PMID: 36050306 PMC9434547

[47] Kamel KS Oh MS Halperin ML . L-lactic acidosis: pathophysiology, classification, and causes; emphasis on biochemical and metabolic basis. Kidney Int. (2020) 97:75–88. doi: 10.1016/j.kint.2019.08.023, PMID: 31784049

[48] Khacho M Tarabay M Patten D Khacho P MacLaurin JG Guadagno J . Acidosis overrides oxygen deprivation to maintain mitochondrial function and cell survival. Nat Commun. (2014) 5:3550. doi: 10.1038/ncomms4550, PMID: 24686499 PMC3988820

[49] Certo M Tsai C-H Pucino V Ho P-C Mauro C . Lactate modulation of immune responses in inflammatory versus tumour microenvironments. Nat Rev Immunol. (2021) 21:151–61. doi: 10.1038/s41577-020-0406-2, PMID: 32839570

[50] Llibre A Kucuk S Gope A Certo M Mauro C . Lactate: A key regulator of the immune response. Immunity. (2025) 58:535–54. doi: 10.1016/j.immuni.2025.02.008, PMID: 40073846

[51] Noe JT Rendon BE Geller AE Conroy LR Morrissey SM Young LEA . Lactate supports a metabolic-epigenetic link in macrophage polarization. Sci Adv. (2021) 7:eabi8602. doi: 10.1126/sciadv.abi8602, PMID: 34767443 PMC8589316

[52] Liu X Li S Cui Q Guo B Ding W Liu J . Activation of GPR81 by lactate drives tumour-induced cachexia. Nat Metab. (2024) 6:708–23. doi: 10.1038/s42255-024-01011-0, PMID: 38499763 PMC11052724

[53] Baltan S . Can lactate serve as an energy substrate for axons in good times and in bad, in sickness and in health? Metab Brain Dis. (2015) 30:25–30. doi: 10.1007/s11011-014-9595-3, PMID: 25034458 PMC4297510

[54] Rabinowitz JD Enerbäck S . Lactate: the ugly duckling of energy metabolism. Nat Metab. (2020) 2:566–71. doi: 10.1038/s42255-020-0243-4, PMID: 32694798 PMC7983055

[55] Blevins HM Xu Y Biby S Zhang S . The NLRP3 inflammasome pathway: A review of mechanisms and inhibitors for the treatment of inflammatory diseases. Front Aging Neurosci. (2022) 14:879021. doi: 10.3389/fnagi.2022.879021, PMID: 35754962 PMC9226403

[56] Lin H-C Chen Y-J Wei Y-H Lin H-A Chen C-C Liu T-F . Lactic acid fermentation is required for NLRP3 inflammasome activation. Front Immunol. (2021) 12:630380. doi: 10.3389/fimmu.2021.630380, PMID: 33854503 PMC8039150

[57] Adeva-Andany M López-Ojén M Funcasta-Calderón R Ameneiros-Rodríguez E Donapetry-García C Vila-Altesor M . Comprehensive review on lactate metabolism in human health. Mitochondrion. (2014) 17:76–100. doi: 10.1016/j.mito.2014.05.007, PMID: 24929216

[58] Fang Y Li Z Yang L Li W Wang Y Kong Z . Emerging roles of lactate in acute and chronic inflammation. Cell Commun Signal. (2024) 22:276. doi: 10.1186/s12964-024-01624-8, PMID: 38755659 PMC11097486

[59] Liao Z Chen B Yang T Zhang W Mei Z . Lactylation modification in cardio-cerebral diseases: A state-of-the-art review. Ageing Res Rev. (2025) 104:102631. doi: 10.1016/j.arr.2024.102631, PMID: 39647583

[60] Li R Yang Y Wang H Zhang T Duan F Wu K . Lactate and lactylation in the brain: current progress and perspectives. Cell Mol Neurobiol. (2023) 43:2541–55. doi: 10.1007/s10571-023-01335-7, PMID: 36928470 PMC11410153

[61] Wannemacher RW Pace JG Beall RA Dinterman RE Petrella VJ Neufeld HA . Role of the liver in regulation of ketone body production during sepsis. J Clin Invest. (1979) 64:1565–72. doi: 10.1172/JCI109617, PMID: 500825 PMC371309

[62] Vandewalle J Libert C . Sepsis: a failing starvation response. Trends Endocrinol Metab. (2022) 33:292–304. doi: 10.1016/j.tem.2022.01.006, PMID: 35181202

[63] Nelson AB Queathem ED Puchalska P Crawford PA . Metabolic Messengers: ketone bodies. Nat Metab. (2023) 5:2062–74. doi: 10.1038/s42255-023-00935-3, PMID: 38092961

[64] Rahmel T Hübner M Koos B Wolf A Willemsen K-M Strauß G . Impact of carbohydrate-reduced nutrition in septic patients on ICU: study protocol for a prospective randomised controlled trial. BMJ Open. (2020) 10:e038532. doi: 10.1136/bmjopen-2020-038532, PMID: 32641340 PMC7348645

[65] Stanzani G Duchen MR Singer M . The role of mitochondria in sepsis-induced cardiomyopathy. Biochim Biophys Acta Mol Basis Dis. (2019) 1865:759–73. doi: 10.1016/j.bbadis.2018.10.011, PMID: 30342158

[66] Giri B Dey S Das T Sarkar M Banerjee J Dash SK . Chronic hyperglycemia mediated physiological alteration and metabolic distortion leads to organ dysfunction, infection, cancer progression and other pathophysiological consequences: An update on glucose toxicity. BioMed Pharmacother. (2018) 107:306–28. doi: 10.1016/j.biopha.2018.07.157, PMID: 30098549

[67] Rivas AM Nugent K . Hyperglycemia, insulin, and insulin resistance in sepsis. Am J Med Sci. (2021) 361:297–302. doi: 10.1016/j.amjms.2020.11.007, PMID: 33500122

[68] Lee AK Kim DH Bang E Choi YJ Chung HY . β-hydroxybutyrate suppresses lipid accumulation in aged liver through GPR109A-mediated signaling. Aging Dis. (2020) 11:777–90. doi: 10.14336/AD.2019.0926, PMID: 32765945 PMC7390524

[69] Youm Y-H Nguyen KY Grant RW Goldberg EL Bodogai M Kim D . The ketone metabolite β-hydroxybutyrate blocks NLRP3 inflammasome-mediated inflammatory disease. Nat Med. (2015) 21:263–9. doi: 10.1038/nm.3804, PMID: 25686106 PMC4352123

[70] Hao H Cao L Jiang C Che Y Zhang S Takahashi S . Farnesoid X receptor regulation of the NLRP3 inflammasome underlies cholestasis-associated sepsis. Cell Metab. (2017) 25:856–867.e5. doi: 10.1016/j.cmet.2017.03.007, PMID: 28380377 PMC6624427

[71] Danielski LG Giustina AD Bonfante S Barichello T Petronilho F . The NLRP3 inflammasome and its role in sepsis development. Inflammation. (2020) 43:24–31. doi: 10.1007/s10753-019-01124-9, PMID: 31741197

[72] Soni S Skow RJ Foulkes S Haykowsky MJ Dyck JRB . Therapeutic potential of ketone bodies on exercise intolerance in heart failure: looking beyond the heart. Cardiovasc Res. (2025) 121:230–40. doi: 10.1093/cvr/cvaf004, PMID: 39825790 PMC12012446

[73] Greco T Glenn TC Hovda DA Prins ML . Ketogenic diet decreases oxidative stress and improves mitochondrial respiratory complex activity. J Cereb Blood Flow Metab. (2016) 36:1603–13. doi: 10.1177/0271678X15610584, PMID: 26661201 PMC5012517

[74] Rojas-Morales P Pedraza-Chaverri J Tapia E . Ketone bodies, stress response, and redox homeostasis. Redox Biol. (2020) 29:101395. doi: 10.1016/j.redox.2019.101395, PMID: 31926621 PMC6911969

[75] Kim DY Davis LM Sullivan PG Maalouf M Simeone TA van Brederode J . Ketone bodies are protective against oxidative stress in neocortical neurons. J Neurochem. (2007) 101:1316–26. doi: 10.1111/j.1471-4159.2007.04483.x, PMID: 17403035

[76] Aromiwura AA Gouwens KR Nguyen DC Sztukowska M Didelot L Kalra DK . Ketone bodies in the regulation of myocardial perfusion in cardiovascular disease: metabolic and vasodilatory effects. Int J Mol Sci. (2025) 26:4856. doi: 10.3390/ijms26104856, PMID: 40429996 PMC12111909

[77] Yang H Shan W Zhu F Wu J Wang Q . Ketone bodies in neurological diseases: focus on neuroprotection and underlying mechanisms. Front Neurol. (2019) 10:585. doi: 10.3389/fneur.2019.00585, PMID: 31244753 PMC6581710

[78] Jensen NJ Wodschow HZ Nilsson M Rungby J . Effects of ketone bodies on brain metabolism and function in neurodegenerative diseases. Int J Mol Sci. (2020) 21:8767. doi: 10.3390/ijms21228767, PMID: 33233502 PMC7699472

[79] Nogueira AC Kawabata V Biselli P Lins MH Valeri C Seckler M . Changes in plasma free fatty acid levels in septic patients are associated with cardiac damage and reduction in heart rate variability. Shock. (2008) 29:342–8. doi: 10.1097/shk.0b013e31815abbc6, PMID: 18000476

[80] Henderson GC . Plasma free fatty acid concentration as a modifiable risk factor for metabolic disease. Nutrients. (2021) 13:2590. doi: 10.3390/nu13082590, PMID: 34444750 PMC8402049

[81] Bhathena SJ . Relationship between fatty acids and the endocrine system. Biofactors. (2000) 13:35–9. doi: 10.1002/biof.5520130107, PMID: 11237196

[82] Grabner GF Xie H Schweiger M Zechner R . Lipolysis: cellular mechanisms for lipid mobilization from fat stores. Nat Metab. (2021) 3:1445–65. doi: 10.1038/s42255-021-00493-6, PMID: 34799702

[83] Nielsen TS Jessen N Jørgensen JOL Møller N Lund S . Dissecting adipose tissue lipolysis: molecular regulation and implications for metabolic disease. J Mol Endocrinol. (2014) 52:R199–222. doi: 10.1530/JME-13-0277, PMID: 24577718

[84] Sancar G Liu S Gasser E Alvarez JG Moutos C Kim K . FGF1 and insulin control lipolysis by convergent pathways. Cell Metab. (2022) 34:171–183.e6. doi: 10.1016/j.cmet.2021.12.004, PMID: 34986332 PMC8863067

[85] Kolyva AS Zolota V Mpatsoulis D Skroubis G Solomou EE Habeos IG . The role of obesity in the immune response during sepsis. Nutr Diabetes. (2014) 4:e137. doi: 10.1038/nutd.2014.34, PMID: 25244356 PMC4183975

[86] Karampela I Christodoulatos GS Dalamaga M . The role of adipose tissue and adipokines in sepsis: inflammatory and metabolic considerations, and the obesity paradox. Curr Obes Rep. (2019) 8:434–57. doi: 10.1007/s13679-019-00360-2, PMID: 31637623

[87] Hu D Sheeja Prabhakaran H Zhang Y-Y Luo G He W Liou Y-C . Mitochondrial dysfunction in sepsis: mechanisms and therapeutic perspectives. Crit Care. (2024) 28:292. doi: 10.1186/s13054-024-05069-w, PMID: 39227925 PMC11373266

[88] Paumelle R Haas JT Hennuyer N Baugé E Deleye Y Mesotten D . Hepatic PPARα is critical in the metabolic adaptation to sepsis. J Hepatol. (2019) 70:963–73. doi: 10.1016/j.jhep.2018.12.037, PMID: 30677458 PMC6774768

[89] Kim MS Sweeney TR Shigenaga JK Chui LG Moser A Grunfeld C . Tumor necrosis factor and interleukin 1 decrease RXRalpha, PPARalpha, PPARgamma, LXRalpha, and the coactivators SRC-1, PGC-1alpha, and PGC-1beta in liver cells. Metabolism. (2007) 56:267–79. doi: 10.1016/j.metabol.2006.10.007, PMID: 17224343 PMC2700944

[90] Jin D Sun J Huang J He Y Yu A Yu X . TNF-α reduces g0s2 expression and stimulates lipolysis through PPAR-γ inhibition in 3T3-L1 adipocytes. Cytokine. (2014) 69:196–205. doi: 10.1016/j.cyto.2014.06.005, PMID: 24993166

[91] Pan J Zhang L Li D Li Y Lu M Hu Y . Hypoxia-inducible factor-1: Regulatory mechanisms and drug therapy in myocardial infarction. Eur J Pharmacol. (2024) 963:176277. doi: 10.1016/j.ejphar.2023.176277, PMID: 38123007

[92] Ruan H Zhang Q Zhang Y-P Li S-S Ran X . Unraveling the role of HIF-1α in sepsis: from pathophysiology to potential therapeutics-a narrative review. Crit Care. (2024) 28:100. doi: 10.1186/s13054-024-04885-4, PMID: 38539163 PMC10976824

[93] Mansfield KD Guzy RD Pan Y Young RM Cash TP Schumacker PT . Mitochondrial dysfunction resulting from loss of cytochrome c impairs cellular oxygen sensing and hypoxic HIF-alpha activation. Cell Metab. (2005) 1:393–9. doi: 10.1016/j.cmet.2005.05.003, PMID: 16054088 PMC3141219

[94] Shen H Ojo OA Ding H Mullen LJ Xing C Hossain MI . HIF1α-regulated glycolysis promotes activation-induced cell death and IFN-γ induction in hypoxic T cells. Nat Commun. (2024) 15:9394. doi: 10.1038/s41467-024-53593-8, PMID: 39477954 PMC11526104

[95] Kierans SJ Taylor CT . Regulation of glycolysis by the hypoxia-inducible factor (HIF): implications for cellular physiology. J Physiol. (2021) 599:23–37. doi: 10.1113/JP280572, PMID: 33006160

[96] Romney SJ Newman BS Thacker C Leibold EA . HIF-1 regulates iron homeostasis in Caenorhabditis elegans by activation and inhibition of genes involved in iron uptake and storage. PloS Genet. (2011) 7:e1002394. doi: 10.1371/journal.pgen.1002394, PMID: 22194696 PMC3240588

[97] Tang Y-Y Wang D-C Wang Y-Q Huang A-F Xu W-D . Emerging role of hypoxia-inducible factor-1α in inflammatory autoimmune diseases: A comprehensive review. Front Immunol. (2022) 13:1073971. doi: 10.3389/fimmu.2022.1073971, PMID: 36761171 PMC9905447

[98] McGettrick AF O’Neill LAJ . The role of HIF in immunity and inflammation. Cell Metab. (2020) 32:524–36. doi: 10.1016/j.cmet.2020.08.002, PMID: 32853548

[99] Panwar V Singh A Bhatt M Tonk RK Azizov S Raza AS . Multifaceted role of mTOR (mammalian target of rapamycin) signaling pathway in human health and disease. Signal Transduct Target Ther. (2023) 8:375. doi: 10.1038/s41392-023-01608-z, PMID: 37779156 PMC10543444

[100] Cheng Mm W Long Y Wang H Han Mm W Zhang J Cui N . Role of the mTOR signalling pathway in human sepsis-induced myocardial dysfunction. Can J Cardiol. (2019) 35:875–83. doi: 10.1016/j.cjca.2019.03.022, PMID: 31292086

[101] Dibble CC Cantley LC . Regulation of mTORC1 by PI3K signaling. Trends Cell Biol. (2015) 25:545–55. doi: 10.1016/j.tcb.2015.06.002, PMID: 26159692 PMC4734635

[102] Haythorne E Lloyd M Walsby-Tickle J Tarasov AI Sandbrink J Portillo I . Altered glycolysis triggers impaired mitochondrial metabolism and mTORC1 activation in diabetic β-cells. Nat Commun. (2022) 13:6754. doi: 10.1038/s41467-022-34095-x, PMID: 36376280 PMC9663558

[103] Bar-Tana J . mTORC1 syndrome (TorS): unified paradigm for diabetes/metabolic syndrome. Trends Endocrinol Metab. (2023) 34:135–45. doi: 10.1016/j.tem.2023.01.001, PMID: 36717300

[104] Dodd KM Yang J Shen MH Sampson JR Tee AR . mTORC1 drives HIF-1α and VEGF-A signalling via multiple mechanisms involving 4E-BP1, S6K1 and STAT3. Oncogene. (2015) 34:2239–50. doi: 10.1038/onc.2014.164, PMID: 24931163 PMC4172452

[105] Cam H Easton JB High A Houghton PJ . mTORC1 signaling under hypoxic conditions is controlled by ATM-dependent phosphorylation of HIF-1α. Mol Cell. (2010) 40:509–20. doi: 10.1016/j.molcel.2010.10.030, PMID: 21095582 PMC3004768

[106] Gremke N Polo P Dort A Schneikert J Elmshäuser S Brehm C . mTOR-mediated cancer drug resistance suppresses autophagy and generates a druggable metabolic vulnerability. Nat Commun. (2020) 11:4684. doi: 10.1038/s41467-020-18504-7, PMID: 32943635 PMC7499183

[107] Deleyto-Seldas N Efeyan A . The mTOR-autophagy axis and the control of metabolism. Front Cell Dev Biol. (2021) 9:655731. doi: 10.3389/fcell.2021.655731, PMID: 34277603 PMC8281972

[108] Weichhart T Hengstschläger M Linke M . Regulation of innate immune cell function by mTOR. Nat Rev Immunol. (2015) 15:599–614. doi: 10.1038/nri3901, PMID: 26403194 PMC6095456

[109] Lin X Du Y Kan S Chen J Yin Y Li L . Sustained mTORC1 activation in activated T cells impairs vaccine responses in older individuals. Sci Adv. (2025) 11:eadt4881. doi: 10.1126/sciadv.adt4881, PMID: 40249803 PMC12007566

[110] Hardie DG Ross FA Hawley SA . AMPK: a nutrient and energy sensor that maintains energy homeostasis. Nat Rev Mol Cell Biol. (2012) 13:251–62. doi: 10.1038/nrm3311, PMID: 22436748 PMC5726489

[111] Huang J Liu K Zhu S Xie M Kang R Cao L . AMPK regulates immunometabolism in sepsis. Brain Behav Immun. (2018) 72:89–100. doi: 10.1016/j.bbi.2017.11.003, PMID: 29109024

[112] Xiang H-C Lin L-X Hu X-F Zhu H Li H-P Zhang R-Y . AMPK activation attenuates inflammatory pain through inhibiting NF-κB activation and IL-1β expression. J Neuroinflamm. (2019) 16:34. doi: 10.1186/s12974-019-1411-x, PMID: 30755236 PMC6373126

[113] O’Neill HM Holloway GP Steinberg GR . AMPK regulation of fatty acid metabolism and mitochondrial biogenesis: implications for obesity. Mol Cell Endocrinol. (2013) 366:135–51. doi: 10.1016/j.mce.2012.06.019, PMID: 22750049

[114] O’Neill LAJ Hardie DG . Metabolism of inflammation limited by AMPK and pseudo-starvation. Nature. (2013) 493:346–55. doi: 10.1038/nature11862, PMID: 23325217

[115] Marin TL Gongol B Zhang F Martin M Johnson DA Xiao H . AMPK promotes mitochondrial biogenesis and function by phosphorylating the epigenetic factors DNMT1, RBBP7, and HAT1. Sci Signal. (2017) 10:eaaf7478. doi: 10.1126/scisignal.aaf7478, PMID: 28143904 PMC5830108

[116] Park S Jeon JH Min BK Ha CM Thoudam T Park BY . Role of the pyruvate dehydrogenase complex in metabolic remodeling: differential pyruvate dehydrogenase complex functions in metabolism. Diabetes Metab J. (2018) 42:270–81. doi: 10.4093/dmj.2018.0101, PMID: 30136450 PMC6107359

[117] Zeng Z Huang Q Mao L Wu J An S Chen Z . The pyruvate dehydrogenase complex in sepsis: metabolic regulation and targeted therapy. Front Nutr. (2021) 8:783164. doi: 10.3389/fnut.2021.783164, PMID: 34970577 PMC8712327

[118] Zheng D Liwinski T Elinav E . Inflammasome activation and regulation: toward a better understanding of complex mechanisms. Cell Discov. (2020) 6:36. doi: 10.1038/s41421-020-0167-x, PMID: 32550001 PMC7280307

[119] Long DL McCall CE Poole LB . Glutathionylation of pyruvate dehydrogenase complex E2 and inflammatory cytokine production during acute inflammation are magnified by mitochondrial oxidative stress. Redox Biol. (2023) 65:102841. doi: 10.1016/j.redox.2023.102841, PMID: 37566945 PMC10440583

[120] Galley HF . Oxidative stress and mitochondrial dysfunction in sepsis. Br J Anaesth. (2011) 107:57–64. doi: 10.1093/bja/aer093, PMID: 21596843

[121] Quinn WJ Jiao J TeSlaa T Stadanlick J Wang Z Wang L . Lactate limits T cell proliferation via the NAD(H) redox state. Cell Rep. (2020) 33:108500. doi: 10.1016/j.celrep.2020.108500, PMID: 33326785 PMC7830708

[122] Go S Kramer TT Verhoeven AJ Oude Elferink RPJ Chang J-C . The extracellular lactate-to-pyruvate ratio modulates the sensitivity to oxidative stress-induced apoptosis via the cytosolic NADH/NAD+ redox state. Apoptosis. (2021) 26:38–51. doi: 10.1007/s10495-020-01648-8, PMID: 33230593 PMC7902596

[123] Lei C Guo X Zhang M Zhou X Ding N Ren J . Regulating the metabolic flux of pyruvate dehydrogenase bypass to enhance lipid production in Saccharomyces cerevisiae. Commun Biol. (2024) 7:1399. doi: 10.1038/s42003-024-07103-7, PMID: 39462103 PMC11513081

[124] Guo B Zhang F Yin Y Ning X Zhang Z Meng Q . Post-translational modifications of pyruvate dehydrogenase complex in cardiovascular disease. iScience. (2024) 27:110633. doi: 10.1016/j.isci.2024.110633, PMID: 39224515 PMC11367490

[125] Zangari J Petrelli F Maillot B Martinou J-C . The multifaceted pyruvate metabolism: role of the mitochondrial pyruvate carrier. Biomolecules. (2020) 10:1068. doi: 10.3390/biom10071068, PMID: 32708919 PMC7407832

[126] Kim M-J Lee H Chanda D Thoudam T Kang H-J Harris RA . The role of pyruvate metabolism in mitochondrial quality control and inflammation. Mol Cells. (2023) 46:259–67. doi: 10.14348/molcells.2023.2128, PMID: 36756776 PMC10183795

[127] He Z Zhang J Xu Y Fine EJ Suomivuori C-M Dror RO . Structure of mitochondrial pyruvate carrier and its inhibition mechanism. Nature. (2025) 641:250–7. doi: 10.1038/s41586-025-08667-y, PMID: 40044865 PMC12043432

[128] Webb BA Forouhar F Szu F-E Seetharaman J Tong L Barber DL . Structures of human phosphofructokinase-1 and atomic basis of cancer-associated mutations. Nature. (2015) 523:111–4. doi: 10.1038/nature14405, PMID: 25985179 PMC4510984

[129] Lynch EM Hansen H Salay L Cooper M Timr S Kollman JM . Structural basis for allosteric regulation of human phosphofructokinase-1. Nat Commun. (2024) 15:7323. doi: 10.1038/s41467-024-51808-6, PMID: 39183237 PMC11345425

[130] Xiao M Liu D Xu Y Mao W Li W . Role of PFKFB3-driven glycolysis in sepsis. Ann Med. (2023) 55:1278–89. doi: 10.1080/07853890.2023.2191217, PMID: 37199341 PMC10198010

[131] Ni X Lu C-P Xu G-Q Ma J-J . Transcriptional regulation and post-translational modifications in the glycolytic pathway for targeted cancer therapy. Acta Pharmacol Sin. (2024) 45:1533–55. doi: 10.1038/s41401-024-01264-1, PMID: 38622288 PMC11272797

[132] Bhol NK Bhanjadeo MM Singh AK Dash UC Ojha RR Majhi S . The interplay between cytokines, inflammation, and antioxidants: mechanistic insights and therapeutic potentials of various antioxidants and anti-cytokine compounds. BioMed Pharmacother. (2024) 178:117177. doi: 10.1016/j.biopha.2024.117177, PMID: 39053423

[133] Yi W Clark PM Mason DE Keenan MC Hill C Goddard WA . PFK1 glycosylation is a key regulator of cancer cell growth and central metabolic pathways. Science. (2012) 337:975–80. doi: 10.1126/science.1222278, PMID: 22923583 PMC3534962

[134] Schilperoort M Ngai D Katerelos M Power DA Tabas I . PFKFB2-mediated glycolysis promotes lactate-driven continual efferocytosis by macrophages. Nat Metab. (2023) 5:431–44. doi: 10.1038/s42255-023-00736-8, PMID: 36797420 PMC10050103

[135] Yi W Clark PM Mason DE Keenan MC Hill C Goddard WA . Phosphofructokinase 1 glycosylation regulates cell growth and metabolism. Science. (2012) 337:975–80. doi: 10.1126/science.1222278, PMID: 22923583 PMC3534962

[136] Hoque R Farooq A Ghani A Gorelick F Mehal WZ . Lactate reduces liver and pancreatic injury in Toll-like receptor- and inflammasome-mediated inflammation via GPR81-mediated suppression of innate immunity. Gastroenterology. (2014) 146:1763–74. doi: 10.1053/j.gastro.2014.03.014, PMID: 24657625 PMC4104305

[137] Stüve P Godoy GJ Ferreyra FN Hellriegel F Boukhallouk F Kao Y-S . ACC1 is a dual metabolic-epigenetic regulator of Treg stability and immune tolerance. Mol Metab. (2025) 94:102111. doi: 10.1016/j.molmet.2025.102111, PMID: 39929287 PMC11893314

[138] Hunkeler M Hagmann A Stuttfeld E Chami M Guri Y Stahlberg H . Structural basis for regulation of human acetyl-CoA carboxylase. Nature. (2018) 558:470–4. doi: 10.1038/s41586-018-0201-4, PMID: 29899443

[139] Li R Meng M Chen Y Pan T Li Y Deng Y . ATP-citrate lyase controls endothelial gluco-lipogenic metabolism and vascular inflammation in sepsis-associated organ injury. Cell Death Dis. (2023) 14:401. doi: 10.1038/s41419-023-05932-8, PMID: 37414769 PMC10325983

[140] Wang Y Yu W Li S Guo D He J Wang Y . Acetyl-coA carboxylases and diseases. Front Oncol. (2022) 12:836058. doi: 10.3389/fonc.2022.836058, PMID: 35359351 PMC8963101

[141] Batchuluun B Pinkosky SL Steinberg GR . Lipogenesis inhibitors: therapeutic opportunities and challenges. Nat Rev Drug Discov. (2022) 21. doi: 10.1038/s41573-021-00367-2, PMID: 35031766 PMC8758994

[142] Zhao J Wu Y Rong X Zheng C Guo J . Anti-lipolysis induced by insulin in diverse pathophysiologic conditions of adipose tissue. Diabetes Metab Syndrome Obesity: Targets Ther. (2020) 13. doi: 10.2147/DMSO.S250699, PMID: 32494174 PMC7227813

[143] Morigny P Houssier M Mouisel E Langin D . Adipocyte lipolysis and insulin resistance. Biochimie. (2016) 125. doi: 10.1016/j.biochi.2015.10.024, PMID: 26542285

[144] Wang Y Che M Xin J Zheng Z Li J Zhang S . The role of IL-1β and TNF-α in intervertebral disc degeneration. BioMed Pharmacother. (2020) 131:110660. doi: 10.1016/j.biopha.2020.110660, PMID: 32853910

[145] Davis MS Solbiati J Cronan JE . Overproduction of acetyl-CoA carboxylase activity increases the rate of fatty acid biosynthesis in Escherichia coli. J Biol Chem. (2000) 275:28593–8. doi: 10.1074/jbc.M004756200, PMID: 10893421

[146] Lipke K Kubis-Kubiak A Piwowar A . Molecular mechanism of lipotoxicity as an interesting aspect in the development of pathological states-current view of knowledge. Cells. (2022) 11:844. doi: 10.3390/cells11050844, PMID: 35269467 PMC8909283

[147] Dong C . Cytokine regulation and function in T cells. Annu Rev Immunol. (2021) 39:51–76. doi: 10.1146/annurev-immunol-061020-053702, PMID: 33428453

[148] He W Xiao K Xu J Guan W Xie S Wang K . Recurrent sepsis exacerbates CD4+ T cell exhaustion and decreases antiviral immune responses. Front Immunol. (2021) 12:627435. doi: 10.3389/fimmu.2021.627435, PMID: 33717146 PMC7946831

[149] Yi JS Cox MA Zajac AJ . T-cell exhaustion: characteristics, causes and conversion. Immunology. (2010) 129:474–81. doi: 10.1111/j.1365-2567.2010.03255.x, PMID: 20201977 PMC2842494

[150] Jung J Zeng H Horng T . Metabolism as a guiding force for immunity. Nat Cell Biol. (2019) 21:85–93. doi: 10.1038/s41556-018-0217-x, PMID: 30602764

[151] Pearce EL Pearce EJ . Metabolic pathways in immune cell activation and quiescence. Immunity. (2013) 38:633–43. doi: 10.1016/j.immuni.2013.04.005, PMID: 23601682 PMC3654249

[152] Sun X-F Luo W-C Huang S-Q Zheng Y-J Xiao L Zhang Z-W . Immune-cell signatures of persistent inflammation, immunosuppression, and catabolism syndrome after sepsis. Med. (2025) 6:100569. doi: 10.1016/j.medj.2024.12.003, PMID: 39824181

[153] Pearce EL . Metabolism in T cell activation and differentiation. Curr Opin Immunol. (2010) 22:314–20. doi: 10.1016/j.coi.2010.01.018, PMID: 20189791 PMC4486663

[154] De Leon-Oliva D González-Prieto P De Castro-Martinez P Boaru DL Laguna-Hernández P Fraile-Martinez O . Revisiting the biological role of the Warburg effect: Evolving perspectives on cancer metabolism. Pathol - Res Pract. (2025), 156151. doi: 10.1016/j.prp.2025.156151, PMID: 40743579

[155] Martin MD Badovinac VP Griffith TS . CD4 T cell responses and the sepsis-induced immunoparalysis state. Front Immunol. (2020) 11:1364. doi: 10.3389/fimmu.2020.01364, PMID: 32733454 PMC7358556

[156] Assis PA Allen RM Schaller MA Kunkel SL Bermick JR . Metabolic reprogramming and dysregulated IL-17 production impairs CD4 T cell function post sepsis. iScience. (2024) 27:110114. doi: 10.1016/j.isci.2024.110114, PMID: 39015145 PMC11251092

[157] Wu H Zhao X Hochrein SM Eckstein M Gubert GF Knöpper K . Mitochondrial dysfunction promotes the transition of precursor to terminally exhausted T cells through HIF-1α-mediated glycolytic reprogramming. Nat Commun. (2023) 14:6858. doi: 10.1038/s41467-023-42634-3, PMID: 37891230 PMC10611730

[158] Baessler A Vignali DAA . T cell exhaustion. Annu Rev Immunol. (2024) 42:179–206. doi: 10.1146/annurev-immunol-090222-110914, PMID: 38166256

[159] Oh SA Wu D-C Cheung J Navarro A Xiong H Cubas R . PD-L1 expression by dendritic cells is a key regulator of T-cell immunity in cancer. Nat Cancer. (2020) 1:681–91. doi: 10.1038/s43018-020-0075-x, PMID: 35122038

[160] Ibañez-Vega J Vilchez C Jimenez K Guevara C Burgos PI Naves R . Cellular and molecular regulation of the programmed death-1/programmed death ligand system and its role in multiple sclerosis and other autoimmune diseases. J Autoimmun. (2021) 123:102702. doi: 10.1016/j.jaut.2021.102702, PMID: 34311143

[161] Boussiotis VA Patsoukis N . Effects of PD-1 signaling on immunometabolic reprogramming. Immunometabolism. (2022) 4:e220007. doi: 10.20900/immunometab20220007, PMID: 35371563 PMC8975241

[162] Liu R Li H-F Li S . PD-1-mediated inhibition of T cell activation: Mechanisms and strategies for cancer combination immunotherapy. Cell Insight. (2024) 3:100146. doi: 10.1016/j.cellin.2024.100146, PMID: 38425643 PMC10901852

[163] Ogawa T Isik M Wu Z Kurmi K Meng J Cho S . Nutrient control of growth and metabolism through mTORC1 regulation of mRNA splicing. Mol Cell. (2024) 84:4558–4575.e8. doi: 10.1016/j.molcel.2024.10.037, PMID: 39571580 PMC12455899

[164] Cao J Liao S Zeng F Liao Q Luo G Zhou Y . Effects of altered glycolysis levels on CD8+ T cell activation and function. Cell Death Dis. (2023) 14:407. doi: 10.1038/s41419-023-05937-3, PMID: 37422501 PMC10329707

[165] Martins CP New LA O’Connor EC Previte DM Cargill KR Tse IL . Glycolysis inhibition induces functional and metabolic exhaustion of CD4+ T cells in type 1 diabetes. Front Immunol. (2021) 12:669456. doi: 10.3389/fimmu.2021.669456, PMID: 34163475 PMC8216385

[166] Liu S Liao S Liang L Deng J Zhou Y . The relationship between CD4+ T cell glycolysis and their functions. Trends Endocrinol Metab. (2023) 34:345–60. doi: 10.1016/j.tem.2023.03.006, PMID: 37061430

[167] de Azambuja Rodrigues PM Valente RH Brunoro GVF Nakaya HTI Araújo-Pereira M Bozza PT . Proteomics reveals disturbances in the immune response and energy metabolism of monocytes from patients with septic shock. Sci Rep. (2021) 11:15149. doi: 10.1038/s41598-021-94474-0, PMID: 34312428 PMC8313678

[168] Zhang L Shi X Qiu H Liu S Yang T Li X . Protein modification by short-chain fatty acid metabolites in sepsis: a comprehensive review. Front Immunol. (2023) 14:1171834. doi: 10.3389/fimmu.2023.1171834, PMID: 37869005 PMC10587562

[169] Brealey D Karyampudi S Jacques TS Novelli M Stidwill R Taylor V . Mitochondrial dysfunction in a long-term rodent model of sepsis and organ failure. Am J Physiol Regul Integr Comp Physiol. (2004) 286:R491–497. doi: 10.1152/ajpregu.00432.2003, PMID: 14604843

[170] Liao S Chen L Song Z He H . The fate of damaged mitochondrial DNA in the cell. Biochim Biophys Acta (BBA) - Mol Cell Res. (2022) 1869:119233. doi: 10.1016/j.bbamcr.2022.119233, PMID: 35131372

[171] Rong Z Tu P Xu P Sun Y Yu F Tu N . The mitochondrial response to DNA damage. Front Cell Dev Biol. (2021) 9:669379. doi: 10.3389/fcell.2021.669379, PMID: 34055802 PMC8149749

[172] Eyenga P Roussel D Morel J Rey B Romestaing C Gueguen-Chaignon V . Time course of liver mitochondrial function and intrinsic changes in oxidative phosphorylation in a rat model of sepsis. Intensive Care Med Exp. (2018) 6:31. doi: 10.1186/s40635-018-0197-y, PMID: 30187255 PMC6125261

[173] Chen C Zheng H Horwitz EM Ando S Araki K Zhao P . Mitochondrial metabolic flexibility is critical for CD8^+^ T cell antitumor immunity. Sci Adv. (2023) 9:eadf9522. doi: 10.1126/sciadv.adf9522, PMID: 38055827 PMC10699783

[174] Chen W Zhao H Li Y . Mitochondrial dynamics in health and disease: mechanisms and potential targets. Signal Transduct Target Ther. (2023) 8:333. doi: 10.1038/s41392-023-01547-9, PMID: 37669960 PMC10480456

[175] T cells require mitochondria to proliferate, function and generate memory. Nat Immunol. (2025) 26:1227–8. doi: 10.1038/s41590-025-02226-3, PMID: 40696046

[176] Beltra J-C Manne S Abdel-Hakeem MS Kurachi M Giles JR Chen Z . Developmental relationships of four exhausted CD8+ T cell subsets reveals underlying transcriptional and epigenetic landscape control mechanisms. Immunity. (2020) 52:825–841.e8. doi: 10.1016/j.immuni.2020.04.014, PMID: 32396847 PMC8360766

[177] Jenkins E Whitehead T Fellermeyer M Davis SJ Sharma S . The current state and future of T-cell exhaustion research. Oxf Open Immunol. (2023) 4:iqad006. doi: 10.1093/oxfimm/iqad006, PMID: 37554723 PMC10352049

[178] Nagar H Piao S Kim C-S . Role of mitochondrial oxidative stress in sepsis. Acute Crit Care. (2018) 33:65–72. doi: 10.4266/acc.2018.00157, PMID: 31723865 PMC6849061

[179] Matsumoto H Ogura H Shimizu K Ikeda M Hirose T Matsuura H . The clinical importance of a cytokine network in the acute phase of sepsis. Sci Rep. (2018) 8:13995. doi: 10.1038/s41598-018-32275-8, PMID: 30228372 PMC6143513

[180] Fiore A Murray PJ . Tryptophan and indole metabolism in immune regulation. Curr Opin Immunol. (2021) 70:7–14. doi: 10.1016/j.coi.2020.12.001, PMID: 33418116

[181] Mellor AL Munn DH . IDO expression by dendritic cells: tolerance and tryptophan catabolism. Nat Rev Immunol. (2004) 4:762–74. doi: 10.1038/nri1457, PMID: 15459668

[182] Stone TW Williams RO . Modulation of T cells by tryptophan metabolites in the kynurenine pathway. Trends Pharmacol Sci. (2023) 44:442–56. doi: 10.1016/j.tips.2023.04.006, PMID: 37248103

[183] Shi D Wu X Jian Y Wang J Huang C Mo S . USP14 promotes tryptophan metabolism and immune suppression by stabilizing IDO1 in colorectal cancer. Nat Commun. (2022) 13:5644. doi: 10.1038/s41467-022-33285-x, PMID: 36163134 PMC9513055

[184] Jamshed L Debnath A Jamshed S Wish JV Raine JC Tomy GT . An emerging cross-species marker for organismal health: tryptophan-kynurenine pathway. Int J Mol Sci. (2022) 23:6300. doi: 10.3390/ijms23116300, PMID: 35682980 PMC9181223

[185] Stier MT Sewell AE Mwizerwa EL Sim CY Tanner SM Nichols CM . Metabolic adaptations rewire CD4 T cells in a subset-specific manner in human critical illness with and without sepsis. bioRxiv. (2025), 2025.01.27.635146. doi: 10.1101/2025.01.27.635146, PMID: 41540263 PMC12864044

[186] Canè S Geiger R Bronte V . The roles of arginases and arginine in immunity. Nat Rev Immunol. (2025) 25:266–84. doi: 10.1038/s41577-024-01098-2, PMID: 39420221

[187] Schrijver IT Théroude C Roger T . Myeloid-derived suppressor cells in sepsis. Front Immunol. (2019) 10:327. doi: 10.3389/fimmu.2019.00327, PMID: 30873175 PMC6400980

[188] Maulydia M Rehatta NM Soedarmo SM . Effects of glutamine and arginine combination on pro- and anti-inflammatory cytokines. Open Vet J. (2023) 13:613–9. doi: 10.5455/OVJ.2023.v13.i5.14, PMID: 37304602 PMC10257461

[189] Kim MY Jayasinghe R Devenport JM Ritchey JK Rettig MP O’Neal J . A long-acting interleukin-7, rhIL-7-hyFc, enhances CAR T cell expansion, persistence, and anti-tumor activity. Nat Commun. (2022) 13:3296. doi: 10.1038/s41467-022-30860-0, PMID: 35697686 PMC9192727

[190] Bidar F Hamada S Gossez M Coudereau R Lopez J Cazalis M-A . Recombinant human interleukin-7 reverses T cell exhaustion ex vivo in critically ill COVID-19 patients. Ann Intensive Care. (2022) 12:21. doi: 10.1186/s13613-022-00982-1, PMID: 35246776 PMC8896969

[191] de Roquetaillade C Monneret G Gossez M Venet F . IL-7 and its beneficial role in sepsis-induced T lymphocyte dysfunction. Crit Rev Immunol. (2018) 38:433–51. doi: 10.1615/CritRevImmunol.2018027460, PMID: 31002599

[192] Andreu-Ballester JC Cuellar C Garcia-Ballesteros C Pérez-Griera J Amigó V Peiró-Gómez A . Deficit of interleukin 7 in septic patients. Int Immunopharmacol. (2014) 23:73–6. doi: 10.1016/j.intimp.2014.08.015, PMID: 25169828

[193] Wofford JA Wieman HL Jacobs SR Zhao Y Rathmell JC . IL-7 promotes Glut1 trafficking and glucose uptake via STAT5-mediated activation of Akt to support T-cell survival. Blood. (2008) 111:2101–11. doi: 10.1182/blood-2007-06-096297, PMID: 18042802 PMC2234050

[194] Mackall CL Fry TJ Gress RE . Harnessing the biology of IL-7 for therapeutic application. Nat Rev Immunol. (2011) 11:330–42. doi: 10.1038/nri2970, PMID: 21508983 PMC7351348

[195] Szwed A Kim E Jacinto E . Regulation and metabolic functions of mTORC1 and mTORC2. Physiol Rev. (2021) 101:1371–426. doi: 10.1152/physrev.00026.2020, PMID: 33599151 PMC8424549

[196] Bachoo S Gudgeon N Mann R Stavrou V Bishop EL Kelly A . IL-7 promotes integrated glucose and amino acid sensing during homeostatic CD4+ T cell proliferation. Cell Rep. (2025) 44:115199. doi: 10.1016/j.celrep.2024.115199, PMID: 39799568

[197] Pusapati RV Daemen A Wilson C Sandoval W Gao M Haley B . mTORC1-dependent metabolic reprogramming underlies escape from glycolysis addiction in cancer cells. Cancer Cell. (2016) 29:548–62. doi: 10.1016/j.ccell.2016.02.018, PMID: 27052953

[198] Francois B Jeannet R Daix T Walton AH Shotwell MS Unsinger J . Interleukin-7 restores lymphocytes in septic shock: the IRIS-7 randomized clinical trial. JCI Insight. (2018) 3:e98960, 98960. doi: 10.1172/jci.insight.98960, PMID: 29515037 PMC5922293

[199] Liu X Yang M Xu P Du M Li S Shi J . Kynurenine-AhR reduces T-cell infiltration and induces a delayed T-cell immune response by suppressing the STAT1-CXCL9/CXCL10 axis in tuberculosis. Cell Mol Immunol. (2024) 21:1426–40. doi: 10.1038/s41423-024-01230-1, PMID: 39438693 PMC11607402

[200] Mitchell TC Hamid O Smith DC Bauer TM Wasser JS Olszanski AJ . Epacadostat plus pembrolizumab in patients with advanced solid tumors: phase I results from a multicenter, open-label phase I/II trial (ECHO-202/KEYNOTE-037). J Clin Oncol. (2018) 36:3223–30. doi: 10.1200/JCO.2018.78.9602, PMID: 30265610 PMC6225502

[201] Long GV Dummer R Hamid O Gajewski TF Caglevic C Dalle S . Epacadostat plus pembrolizumab versus placebo plus pembrolizumab in patients with unresectable or metastatic melanoma (ECHO-301/KEYNOTE-252): a phase 3, randomised, double-blind study. Lancet Oncol. (2019) 20:1083–97. doi: 10.1016/S1470-2045(19)30274-8, PMID: 31221619

[202] Dreute J Stengel J Becher J van den Borre D Pfisterer M Bartkuhn M . Synergistic targeting of cancer cells through simultaneous inhibition of key metabolic enzymes. Cell Death Differ. (2025). doi: 10.1038/s41418-025-01532-5, PMID: 40550880 PMC12669732

[203] Cheong JE Sun L . Targeting the IDO1/TDO2–KYN–ahR pathway for cancer immunotherapy – challenges and opportunities. Trends Pharmacol Sci. (2018) 39:307–25. doi: 10.1016/j.tips.2017.11.007, PMID: 29254698

